# An Overview and Review of Growth Factors in Wound Healing: Emerging Trends and Innovations

**DOI:** 10.2174/0115733998332692241202072249

**Published:** 2025-05-27

**Authors:** Phool Chandra, Mohd Faizan, Mayur Porwal, Himanshu Sharma, Neetu Sachan

**Affiliations:** 1 Department of Pharmacology, Teerthanker Mahaveer College of Pharmacy, Teerthanker Mahaveer University, Moradabad, Uttar Pradesh, 244101, India;; 2 Department of Pharmaceutical Chemistry, Maharana Pratap College of Pharmacy, Mandhana Kanpur, Uttar Pradesh, 209217, India

**Keywords:** Growth factors, wound healing, PDGF, TGF-β, VEGF, FGF, EGF, tissue repair

## Abstract

Wound healing is a complex process involving various cellular and molecular events aimed at restoring tissue integrity. Growth factors play a pivotal role in orchestrating these events by regulating cell migration, proliferation, differentiation, and extracellular matrix synthesis. Several growth factors have been identified as crucial mediators of wound healing, including PDGF, TGF-β, VEGF, FGF, and EGF. PDGF is released by platelets upon injury and stimulates the migration and proliferation of fibroblasts and smooth muscle cells, promoting the formation of granulation tissue. TGF-β regulates various aspects of wound healing, including inflammation, collagen deposition, and tissue remodeling. VEGF promotes angiogenesis, facilitating the formation of new blood vessels to supply oxygen and nutrients to the healing tissue. FGF stimulates fibroblast proliferation and angiogenesis, contributing to tissue regeneration. EGF promotes the migration and proliferation of epithelial cells, aiding in the re-epithelialization of the wound. These growth factors act in a coordinated manner to promote each phase of wound healing, including hemostasis, inflammation, proliferation, and remodeling. Dysregulation of growth factor expression or signaling can impair the healing process, leading to chronic wounds or excessive scar formation. Understanding the roles of growth factors in wound healing has led to the development of therapeutic strategies aimed at enhancing wound repair.

## 1 INTRODUCTION

Wound healing is a complex process involving hemostasis, inflammation, proliferation, and remodeling, regulated by various growth factors. Problems arise when these factors are imbalanced or deficient. Key growth factors include PDGF (promotes fibroblast function), TGF-β (modulates inflammation and collagen production), EGF (supports re-epithelialization), VEGF (stimulates angiogenesis), FGF (aids cell proliferation), IGF (collagen synthesis), KGF (keratinocyte proliferation), and TNF-α (inflammation control). Factors contributing to imbalance include diabetes, chronic inflammation, aging, nutritional deficiencies, hypoxia, and certain medications. Addressing these imbalances is crucial for managing chronic and difficult-to-heal wounds. Factors responsible for wound healing refer to the coordinated and regulated action of several growth factors, cytokines, and signaling molecules, which are essential for the complex process of wound healing. These substances are vital for the several phases of wound healing, including tissue remodeling, proliferation, and inflammation. These are the primary variables that contribute to the growth and healing of wounds [1]. The correlation between these growth factors highlights their interconnected roles in wound healing. PDGF initiates the recruitment of key cells to the wound site, while TGF-β regulates the transition from inflammation to tissue formation. VEGF ensures that newly forming tissue receives an adequate blood supply, and FGFs enhance the production of the ECM and facilitate cellular proliferation. EGF supports the final stages of wound closure and skin restoration. The synchronized action of these growth factors ensures that wound healing proceeds through the necessary stages of inflammation, proliferation, and remodeling [2]. Essential roles and therapeutic interventions aimed at modulating growth factor activity hold significant potential. Recombinant growth factors, such as PDGF and EGF, have been developed to enhance wound healing in clinical settings, particularly for chronic wounds like diabetic ulcers. Growth factor-containing dressings, which provide a sustained release of these molecules to the wound site, have also shown promise in promoting faster and more effective healing. Additionally, gene therapy approaches that target growth factor signaling pathways could provide more precise and long-lasting interventions for tissue repair [3].

### 1.1 Platelet-derived Growth Factor (PDGF)

Platelet-derived growth factor (PDGF) is a hormone that can cause the proliferation of vascular endothelial cells, vascular smooth muscle fibers, and fibroblasts to help repair damaged blood vessel walls. Multiple cell types' proliferation, differentiation, and activation are regulated by the polypeptide cytokine PDGF [4]. First identified as a platelet-derived growth factor that selectively activates soft tissue and mesenchymal fibroblasts, platelet-derived growth factor isoforms are released by stimulated cells, including macrophages, fibroblasts, and endothelial cells [5]. Numerous wound healing processes, including soft tissue repair, bone repair, and epithelial regeneration, are aided by these isoforms. A further synergistic interaction between PDGF and other growth factors might be the source of some of these actions. The growth factors IGF, FGF, TGF-α, and epidermal growth factor (EGF) are involved in tissue healing. All these growth factors, such as PDGF, belong to distinct families of molecules with similar roles. The growth factors that have been discovered seem to interact in complex and mostly unexamined ways to alter vital physiological processes, including morphogenesis, adult stem cell commitment, wound healing, and embryogenesis. A recent investigation into the potential significance of PDGF in pathological circumstances such as atherosclerosis and autoimmune diseases, as well as normal metabolic working properly, has been launched [6]. PDGFs or platelet-derived growth factors include both small and large substances, such as proteins and hormones [7]. Platelet-derived growth factors (PDGF) are pleasurable chemicals that, in the context of wound recovery processes, strengthen cellular proliferation and differentiation, influence the generation of an extracellular matrix, and expedite tissue regeneration and cellular proliferation (Fig. **1**), [8]. Furthermore, PLT induces keratinocytes to migrate into the wound, resulting in the production of a protective epidermal layer that is caused by its proliferative and cell division-inducing properties. When endothelial damage occurs from close interaction with circulating collagen, platelets, which are ordinarily in a dormant state, become activated [9]. α-granules containing biologically active proteins and PDGF are secreted by activated platelets [10]. These essential elements of hemostasis include leukocyte recruitment, vessel healing, and capillary constriction. PDGF plays a part in all stages of the wound healing process and is mostly produced as a consequence of damage. In acute wounds, it is abundant [11], but in chronic wounds, it is diminished. By binding to transmembrane tyrosine kinase receptors, particularly PDGF receptor-β, it triggers a sequence of internal events that lead to the activation of certain genes, including c-Fos [12]. Like IL-1, PDGF draws neutrophils to the wound site to eradicate germs [13]. Additionally, TGF-inducing monocytes to become macrophages increases the inflammatory response and facilitates tissue cleansing, epithelial cell regrowth, and tissue reorganization. The proliferation of keratinocytes, cell migration, and the reconstruction of the skin's topmost layer are all made possible by EGF, which is essential for the healing of acute wounds. By increasing blood vessel permeability and encouraging the development of new blood vessels (angiogenesis), VEGF speeds up the healing process. CTGF regulates the synthesis of collagen and stimulates angiogenesis, platelet adhesion, and white blood cell motility [14]. The creation and secretion of growth factors by keratinocytes, fibroblasts, and PLT during tissue healing must be emphasized. There are several techniques for creating PC, and each one results in a distinct final product with distinct biological properties, such as different concentrations of growth factors, and possible applications [15]. The lack of consistency and a thorough description of the techniques used makes it difficult to compare the results. When using autologous or allogeneic platelet concentrates, clinicians need to assess the hazards involved, mostly transfusion-transmitted infections. Autologous PLT isolates are recommended by patients since there is no possibility of cross-infection [16]. But in situations when underlying illnesses like cancer, hematologic malignancies, diabetes, *etc.*, may impair the beneficial effects of PC products, it is not recommended. Moreover, there may be differences in quality between autologous preparations of platelet concentrate (PC) and allogeneic preparations from blood banks that meet predetermined standards. Comparing approaches and results is difficult because of this disparity [17]. The use of allogeneic PC has risen because of the significant decrease in viral virus transmission risk caused by the use of NAT in screening blood donors. The crucial role that platelets play is that once platelets are dormant, they circulate the entire body. They are rapidly activated and actively engage in blood clotting and the formation of clots in the bloodstream, whether there is destruction of the vessel walls under certain conditions (like a decrease in nitric oxide levels). In Fig. (**2**) platelets are rich in mRNA and membrane receptors, which facilitate quick healing. PLT aggregates may form and direct protein synthesis is possible as a consequence. PLT cannot establish enduring associations with endothelial cells under ordinary conditions; nevertheless, on pathological or hyperactive endothelial cells, this phenomenon may transpire as a result of insufficient nitric oxide generation.

The inflammation mechanisms that surround endothelial cells intensify when platelets stick to them. This might eventually result in damage, the formation of plaques, and apoptosis of the endothelial cells. Subendothelial matrix proteins, such as collagens and tissue factors expressed on the membrane surface, are visible in endothelium layer lesions. These amino acids have potent coagulation initiation and platelet activation characteristics. Following a vascular lesion, platelets are initially anchored or trapped by the GPIb-IX-V complex interacting with the von Willebrand factor (VWF). VWF interacts with glycoprotein Ib (GPIb) more readily when it attaches to collagen because it changes its form. This touch is not enough to ensure immediate and lasting adhesion. However, it does so *via* activating GPVI, a collagen receptor, which in turn activates integrins IIb3 (GPIIb/IIIa) and 21 (GPIa/IIa). The latter molecules form a powerful bond with elastin and VWF/fibrinogen, which activates GPVI. The feedback messengers TxA2 and ADP are eliminated by the platelets as a result of impulses sent out by the two platelet integrins and GPVI. The platelets distribute as well throughout the network. VWF, fibrinogen, ADP, TxA2, and thrombin commence the process of thrombus development (aggregation) and subsequent stabilization (clot reaction) [17].

Platelets release PDGF at the site of damage, which is complex, and the well-coordinated process of wound healing depends on the signaling molecule PDGF. When blood arteries are damaged or ruptured, platelets and small pieces of blood cells play a vigorous role in reacting and starting the healing process. One of the most important steps in this process is the release of PDGF at the injury site. An explanation of the claim that platelets secrete PDGF at the site of damage is provided below. Activation of platelets undergo activation in response to injury or damage to blood vessels, initiating the subsequent cascade of events. Upon contact with exposed collagen at the site of damage, platelets undergo activation. To cause platelet accumulation, stimulated platelets achieve chemical reactions that draw other platelets to the area. To stop excessive bleeding, platelets clump enough to create a clot that plugs the blood artery break. PDGF is a growth factor that is released by platelets that have been activated. Its release is triggered as a reaction to the injury for tissue repair. PDGF is a potent mitogen, stimulating cell division and proliferation. It selectively focuses on wound-healing cells, specifically fibroblasts and smooth muscle cells. PDGF enhances the migration of fibroblasts, which are cells responsible for forming connective tissue, towards the location of injury. Fibroblasts have a substantial role in the invention of collagen, the primary structural protein found in connective tissues when influenced by PDGF. Stimulating angiogenesis, the emergence of new blood vessels, is another process that PDGF promotes. It is essential to provide the mending tissue with adequate nourishment and oxygen through the repair process. Repair and regeneration of tissues, along with other growth factors, synergistically facilitate tissue regeneration and repair. PDGF promotes the generation of granulation tissue, a transient tissue that facilitates wound healing and supports the development of new blood vessels. PDGF is known for its ability to modify the inflammatory response by regulating inflammatory reactions at the site of injury. It promotes a harmonious equilibrium between inflammation and tissue regeneration, leading to a better-regulated and synchronized healing procedure. Implications for clinical practice advances in methods of treatment may be linked to the understanding of the significance of development factors, including platelet-derived growth factors. Through the use of development factors such as PDGF, which exhibit regenerative properties, platelet-rich plasma (PRP) treatments enhance healing in a variety of medical and surgical settings. As a whole, the body's response to tissue damage is mostly dependent on the production of PDGF at the site of damage. For wound healing, tissue repair, and the return of normal tissue function to occur, PDGF must coordinate the cells' responses. This complex process demonstrates the body's amazing ability to heal and regenerate itself on its own when damaged.

## 2 METHODOLOGY

The review was performed using the databases Medline, Embase, Science Direct, and Scopus, using the keywords “Growth factors, wound healing, PDGF, TGF-β, VEGF, FGF, EGF, tissue repair”. Additional information was gathered from related textbooks, reviews, and documents.

## 3 TRANSFORMING GROWTH FACTOR-BETA (TGF-β)

TGF is a cytokine that is a member of the transforming growth factors superfamily. This subfamily is comprised of many signaling proteins [18], TGF-β1 to 3, with the HGNC symbols TGF-β1, TGF-β2, and TGF-β3. All subtypes of white blood cells create TGF-β protein. Activated TGF- complexes produce serine/threonine kinase complexes that bind to TGF- receptors when mixed with other factors. TGF receptors include subunits of both the type 1 and type 2 receptors. The phosphorylation of type 2 receptor kinase and its subsequent activation of type 1 receptor kinase complying with TGF-binding initiates a signaling cascade. The expression of several target genes involved in the differentiation, chemotaxis, proliferation, and activation of distinct immune cell lines is caused by this mechanism, which sets off a cascade of downstream substrates and regulators [19]. In an inert state, TGF is expressed by a wide variety of cells, including macrophages. In this condition, it combines with LTBP and LAP as two different polypeptides. Proteins called serum proteinases, which include plasmin, aid in releasing active TGF from the complex. The ligand that links CD36 on the surface of the macrophage to the latent TGF-complex is identified as thrombospondin-1 (TSP-1). Activation of plasmin causes macrophages to release more active transforming growth factor-beta (TGF-β). Through endocytosis, macrophages take up IgG-bound latent TGF-complexes made by plasma cells, which release active TGF- into the extracellular fluid. Controlling inflammatory activities, particularly those in the digestive system, is its main function. TGF-β is necessary to direct and regulate T-cell proliferation as well as the process of differentiation of the stem cells [20]. Because of its involvement in immune and stem cell regulation and differentiating themselves, this hormone has been intensively studied in the fields of cancer, autoimmune diseases, and infectious illnesses. Endogenous growth-inhibiting proteins are members of the TGF superfamily. TGF-expression level is often associated with the aggressiveness of many malignancies and does not seem to limit cellular proliferation as TGF would. Therefore, the reality that it may depress the immune system, which further aids in the development of cancer, becomes significant. Its ability to weaken the body's immunity has also been implicated in auto immune illnesses. Nonetheless, this effect may be impacted by other cytokines that are found in the nearby environment (Fig. **3**) [21].

It affects a variety of bodily functions, including the synthesis of extracellular matrix, the generation of proteases, the migration of individual cells in the direction of chemical cues, cell specialization, and proliferation. TGF-β1, TGF-β2, and TGF-β3 are the three isoforms that constitute the transforming growth factor-beta (TGF-β1). Inflammation, angiogenesis, granulation tissue development, and extracellular matrix remodeling are among the activities that TGF-β1 is engaged in [22]. While TGF-β1 knockout mice's full-thickness skin wounds heal nicely at first, by the time they are 3-4 weeks old, they die due to abnormal blood vessel formation and a hyperactive inflammatory response. Therefore, to study wound healing in the absence of TGF-β1 and inflammation, researchers created TGF-β1 knockout mice with an immune-deficient background [23]. The pace of wound healing was not affected significantly between heterozygous and wild- type TGF-β1 mice; however, TGF-β1 animals showed a marked delay in wound healing. All aspects of wound healing were impeded: granulation tissue development was disrupted, inflammatory cell acceptance and fresh blood vessel creation in the wound region were delayed, and cell death was reduced and disorganized. Notably, the lack of TGF-β1 or lymphocytes on their own did not impede wound healing; rather, the lack of both led to a delay. One potential reason is the postponed expression of TGF-β2 and TGF-β3, which occurs when TGF-β1 is not present [23]. TGF-2 contributes to the various stages of wound healing, supporting the development of new blood vessels fibroblasts, and inflammatory cells at the wound site, which increases collagen formation [24]. On the reverse side, TGF-β3 has been shown to promote the growth of new blood vessels and attract fibroblasts and inflammatory cells to the site of destruction, therefore hastening the healing process of wounds. The delayed expression of those variables may be responsible for the observed problems in immunodeficient TGF-β1 knockout mice. Nevertheless, further investigation is needed to validate this theory. Wound-puncture fluids have higher amounts of TGF-β1, KGF, VEGF, HGF, and MMP-1. In either the peripheral blood vessels or hypertrophic scar tissue, patients with burn wounds have increased densities of T cells that produce CD41/TGF-β1. TGF-β1 attracts monocytes to the site of damage by acting as a chemotactic agent for inflamed cells. TGF-β1 is expressed at higher concentrations in fibroids obtained from burn patients [25].

## 4 VASCULAR ENDOTHELIAL GROWTH FACTOR (VEGF)

Cutaneous wounds deviate from acute wound rehabilitation characteristics as an effect of these pathophysiologic techniques. A chronic wound is best described as 'emergent', as it repeatedly fails to heal properly, even though it may not do so quickly. There are between 3 and 6 million Americans who get persistent skin ulcers each year. Venous reflux, hypertension, and type 2 diabetes are the main underlying problems [26]. Almost all serious injuries heal promptly and neatly after a large number of surgical treatments, returning the injured tissue to its usual strength and integrity. Growth factor treatment is a treatment option for lesions that are unable to recover adequately from wet healing. It is thought to activate the missing or malfunctioning components of chronic wounds. When it comes to treating chronic wounds that are impacted by hypoxia and poor blood circulation, the use of a protein called angiogenic growth factor may be beneficial. VEGF is a material that promotes endothelial cell proliferation, draws cells in *via* chemical signals, and causes blood arteries to become permeable [27]. BFGF and TGF are two other known angiogenic growth factors. The fact that VEGF affects angiogenesis, epithelization, and formation of collagen, among other elements of the wound healing process, makes it stand out [28]. This article investigates the role that VEGF angiogenesis plays in the recuperation process of wounds (Fig. **4**).

Human Flt-1 (VEGFR-1) and KDR (VEGFR-2) are receptors of high affinity to which VEGF binds. Seven extracellular domains that resemble immunoglobulins, one transmembrane spanning domain, and one intracellular tyrosine kinase domain distinguish these Type III tyrosine kinases. Despite both their biology and structure being unknown, two more agonists with low affinities and high molecular weight have been designated as VEGF receptors [29]. The surface of both growing and fully formed arteries in the body contains the endothelial proteins Flt-1 and KDR. There is only 37% and 45% similarity across KDR and Flt-1's extracellular and kinase domains, respectively [30]. The development of endothelial cells requires Flk-1, the mouse homolog of KDR, as reported by studies implementing genetic alterations in animal models. Conversely, Flt-1 is essential for blood vessel organization. Membrane ruffling, chemotaxis, and proliferation are preferentially induced by VEGF in endothelial cells expressing KDR, but not in those that express Flt-1 alone. KDR enhances VEGF's mitogenic and chemotactic actions. Although Flt-1's actions are not entirely clear, they most likely include controlling blood artery permeability, drawing neutrophils and macrophages, causing matrix metalloproteinases to form in vascular smooth muscle cells, and promoting the production of anti-apoptotic proteins. Furthermore, a third receptor from the tyrosine kinase family known as VEGFR3 (Flt-4) has been discovered and investigated. Consequently, research has shown that this receptor primarily promotes the development of lymph angiogenesis [31]. The action of VEGF on many elements of the angiogenic process encourages the formation of new blood vessels, a process known as angiogenesis; VEGF contributes to wound healing. This process during wound healing encompasses several stages, including vasodilation, expansion, emigration, and the decomposition of the basement cell membrane by endothelial cells. The creation of capillary tubes ensues, which is followed by the joining of parallel capillary sprouts (loop formation) and, at last, by the production of a new basement membrane. A number of these mechanisms include VEGF [32]. Expansion of blood vessels: one of VEGF's special characteristics is the ability to increase blood flow. Before the discovery of VEGF's amino acid sequence, the protein was known as VPF. Inducing vascular leakage, histamine is not as potent as VEGF. Cyclooxygenase [33] and NOS activity rises after it binds to the KDR site. Prostacyclin and NO both improve vasodilation and permeability of the artery walls. Moreover, stretching and vasodilation may enhance endothelial cells' sensitivity to growth hormones *via* a feedback mechanism, which would raise VEGF synthesis. Degradation of the basement membrane is the production of VEGF, which platelets emit, raising levels of protein in the tissue around it, starting the creation of a blood clot, and ultimately resulting in the production of thrombin and fibrin. Endothelial pregelatinize A is activated by thrombin [34]. The release of gelatinase A (MMP-2) and interstitial collagenase (MMP-1), as well as tissue inhibitor of metalloproteinases (TIMP-1), is directly enhanced by VEGF in endothelial cells [35]. Depending on the dose, VEGF promotes the production of PAI-1 and urokinase- and (uPA and tPA). Additionally, VEGF promotes the vascular smooth muscle cells' synthesis of MMP-1, MMP-3, and MMP-9. The careful balance of enzymatic promoters and inhibitors in the local vascular milieu that VEGF generates serves to allow endothelial migration to occur. Type IV collagen is a protein found in vascular basement membranes, and MMP-2 is capable of breaking it down [36]. Types I-III of collagen are through enzymes broken down by MMP-1 [35]. VEGF isoforms 165, 189, and 206 release physiologically active solubility versions when respective heparin-binding carboxy termini are degraded down by the enzyme plasmin. As a result, enhanced VEGF release can be stimulated by enzymatic activity. The resulting proteolytic environment makes it easier for structural elements like the extracellular matrix and the basement wall to break down, which permits endothelial cells to migrate into the extravascular zone. Endothelial cell migration undergoes the mitotic division when angiogenesis initially originates. Due to the ability of endothelial cells to move and lengthen without splitting, new capillaries can develop *via* budding over four to five days. The next section outlines how VEGF facilitates endothelial migration of cells. The interaction of cell adhesion molecules with the extracellular matrix is known as chemotaxis, and it is a strictly regulated process. The research findings indicate that v5 integrin plays a function in VEGF-induced angiogenesis in the chorioallantois membrane of chicks and rabbits. Furthermore, VEGF stimulates the production of uPA, which is essential for the controlled migration of endothelial cells on vitronectin. However, *in vitro* models show that VEGF increases the expression of the v3 integrin as well as the v5 integrin [37]. Furthermore, osteopontin (OPN), a v3 ligand, is synthesized under the influence of VEGF. OPN demonstrates chemotactic characteristics toward dermal vascular cells, as does thrombin-cleaved OPN [37]. Research is currently focused on the roles represented by these integrins in angiogenesis and wound healing, as well as the possibility that VEGF is selective for a certain integrin pathway. By inducing vascular permeability *via* the actions of the prostacyclin and NO, VEGF stimulates the mobility of endothelial cells during wound healing. When the plasma protein fibrinogen is released into the surrounding environment and ultimately changes into a fibrin gel, endothelial migration is set off. Endothelial cell proliferation is the specific type of mitogen identified as VEGF, which primarily targets endothelial cells. Although the specific molecules responsible for transmitting the mitogenic signal remain unclear, both NO and cGMP certainly play a role in this process. A collagen matrix develops on top of endothelial cells, and VEGF encourages these cells' migration and multiplication, allowing the cells to invade and expand into the matrix below [38]. Furthermore, VEGF inhibits aging and encourages the proliferation of endothelial cells. It inhibits programmed cell death in human endothelial cells and improves the lifespan of endothelial cells by momentarily producing two anti-apoptotic proteins. These proteins are necessary for VEGF to prevent endothelial cells and hematopoietic stem cells from dying as a result of TNF [39]. VEGF may maintain cell attachment and enhance the survival of cells by activating fibronectin and 3 integrin [39]. Although this impact is reliant on vitronectin deposition and enhanced v5 integrin expression, VEGF also inhibits cell death in cells growing on non-supportive, water-repellent surfaces. Caspases 3 and FKHR [40], two pro-apoptotic signals, may be inhibited to suppress apoptosis. Due to their longer absolute lifespans and faster replication rates, endothelial cells play a role in the growth that is induced by VEGF. Further evidence suggests that VEGF simultaneously suppresses signals that lead to cell death and partly mediates the effects of the PI3K/AKT1 or MAP2K1/2/MAPK3/1 pathways on the stimulation of growth of cells and averting cell death [41].

## 5 EPIDERMAL GROWTH FACTOR (EGF)

Various growth factors have a role in the typical healing process of wounds. As a result, growth factors have been suggested as pharmaceuticals to promote wound healing [42]. The 53-amino acid polypeptide known as EGF was isolated by Stanley Cohen in 1962 from the submaxillary gland of a mouse. This remarkable feat served as a springboard for his growth factor research, which brought him the Nobel Prize. The EGF protein family is composed of four proteins: amphiregulin, heparin-binding EGF, TGF-alpha, and EGF. They all have similar structures and biological effects and interact *via* the same receptors on the surface of cells [43]. Skin cell regeneration and wound healing are greatly aided by epidermal growth factor (EGF), which encourages keratinocyte proliferation and migration. Moreover, it promotes granulation tissue formation and fibroblast migration. To activate the tyrosine kinase within the inner domains of the receptor, it must attract and link with cell-surface receptors as a mitogen. This triggers the emergence of DNA and cell division (Fig. **5**) [44]. UVB radiation, IL-33, and ANG II transactivate EGFR-MEK-ERK- or EGFR-PI3K-Akt-dependent keratinocyte migration, survival, and proliferation and type I collagen production is essential for skin wound healing. Furthermore, suppression of neutrophils, monocyte/macrophage/dendritic cells, and lymphocyte infiltration is caused by EGFR activation, which also reduces the production of RANTES, MCP-1, and the CXCR3 ligand IP-10. In addition to inducing ADAM-dependent shedding of EGFR ligands, EGFR activation may also upregulate IL-8 output, which draws neutrophil invasion.

Epidermal growth factor, one of the first introduced by recombinant DNA technology, plays a vital role in wound healing [45]. Following the release of studies that demonstrated minimal benefits in managing chronic wounds, the initial optimism regarding the improved healing of acute surgical wounds through the use of exogenous EGF diminished. Epidermal growth factor (EGF) occurred as the focus shifted to the biological analysis and regulation of the chronic wound environment and the reasons behind compromised wound healing. The effectiveness of growth factors alone has not yet been shown, even though topical growth factor therapy, more especially PDGF from Regards Ethicon in Livingston, UK is currently being utilized to treat chronic wounds, including diabetic foot ulcers [46]. There are now at least three companies in the world marketing EGF-based wound healing medicines for the burn and chronic wound industries. The capacity to promote cell proliferation has been connected to the advancement of epithelial malignancies in addition to its evident influence on the typical healing procedure of wounds [47]. EGF is largely studied for its harmful effects, but it is important to recognize that EGF is also essential for the cellular and molecular mechanisms that lead to proper cutaneous wound healing. The possible outcome of this revived study might be nanomedical growth factor therapies that can improve the process of wound repair by changing the chronic wound microenvironment at a molecular level [47]. The migration and proliferation of keratinocytes during epithelialization are wounded acutely, a protective layer is broken, which causes neutrophils, monocytes, and macrophages to be drawn to the damaged region [48]. The expression of several cytokines and growth factors subsequently causes keratinocytes to become activated. Modifications to the cytoskeleton network and receptors at the cell surface define the engaged phenotype of re-epithelialization [49]. Keratinocyte migration on the wound's edge can repair the damage to the skin's outermost layer, they must first become less attached to the skin's underlying layer and their neighbors. They also need to become more mobile so they can migrate over the newly formed structure. This process is controlled by a series of events that begin with an unraveling of the connections between cells and the substrate that are maintained by desmosomes and hemidesmosomes, respectively. Although the keratinocytes behind the advancing front continue to proliferate, this release makes it easier for them to migrate across the exposed region from the wound's edge [50]. Several regulators play a crucial role in controlling keratinocyte movement and growth throughout the epithelialization process. It is crucial to break down the intercellular link before the migratory activity begins. In the process, desmosomes that are calcium-independent become calcium-dependent, which reduces their adhesive qualities. This is caused by the stimulation of PKC. The transcription factor, a slug (one of the many transcription factors associated with this process), increases the release of keratinocytes by inducing desmosome rupture [51]. The hemidesmosomes connecting the basement membrane to the keratinocyte basal layer must be broken down to allow migration. The basal keratinocytes express a 64-integrin, which in the thick basement cell membrane interacts with laminin-5 (LN5), a particular kind of laminin. The adhesive qualities of hemidesmosomes are influenced by this interaction. The differing preference that integrins show for laminin LN5, especially in its precursor form known as nonproteolytically processed LN5, is one hypothesis that has been put up. The variation that has not been processed shows a strong affinity for 31, whereas the version that has been processed binds only to 64. The amount of integrin that binds to LN5 decreases from 64 to 31 during keratinocyte migration [52]. This process is specifically related to PKC's phosphorylation of serine residues on the four subunits, which increases keratinocyte motility by encouraging hemidesmosome disintegration. Furthermore, the wound environment's levels of EGF and macrophage-stimulating protein affect this PKC-dependent phosphorylation. Migration-associated keratinocytes exhibit increased expression of K6, K16, and K17 keratins, which are thought to augment the viscoelastic properties of migrating cells [53] and are regulated by growth factors present in the wound milieu. Many substances, such as growth factors, cytokines, integrins, keratins, MMPs, chemokines, and extracellular macromolecules, influence keratinocyte migration. Keratinocyte migration and proliferation are stimulated by EGF such as HB-EGF, EGF, and TGF-, which activate EGFR. They also promote the expression of K16 and K6. Our study has shown that GCs may block EGF-induced movement by reducing K6/K16 transcription. In the epidermis of chronic wounds, EGF signaling is not well completed. In contrast to where it accumulates on the outermost layer in the normal epidermis, EGFR in ongoing wounds is mostly found in the cytoplasm of keratinocytes. The protein called galectin-3, which binds to carbohydrates, could be important for the intracellular location of EGFR. Our group has shown that in mice deficient in galectin-3, wound healing is slowed down and EGFR is localized within keratinocyte cells near the wound edge as opposed to on the outer layer of the cell as is the case in wild-type animals with wounds that heal correctly [54]. Furthermore, the epidermis of chronic wounds has been demonstrated to have higher concentrations of ADAM12, a kind of metalloprotease and disintegrant. The inactivation of IGF-binding proteins and HB-EGF has been linked to ADAM12. This shows that one major barrier to persistent wound healing is the failure of keratinocytes close to the wound's bordering to respond to members of the EGF family, even when the members are supplied externally. This might explain why there are certain chronic wounds in individuals when using topically applied EGF does slightly accelerate the healing process [55]. Increased keratinocyte migration and proliferation as a result of that interaction accelerate the healing process of lesions [50]. The proliferation of keratinocytes increases to ensure the presence of enough cells for healing the wound when the topmost layer of the wound, referred to as the migrating epithelial tongue, advances. Keratinocyte proliferation is influenced by the degree of a cell's differentiation, availability of growth agents, and adherence to the substrate. Fully developed keratinocytes in the parabasal layer are incapable of proliferating into new ones; only basal keratinocytes possess this ability [56]. During the epithelialization process, progress influences such as HB-EGF, EGF, TGF, and KGF are primarily responsible for promoting cell proliferation. IGF-1 is an expanding factor that is present in wounds. Studies have shown that it co-activates HB-EGF to promote keratinocyte growth. In transgenic mice that had undergone genetic alteration to boost their production of epidermal granulocyte- macrophage colony-stimulating factor, the cells known as keratinocytes, which are primarily accountable for producing the outer layer of skin, grew faster. Scientific research indicates that this growth factor can accelerate the healing process of wounds [57]. MMPs, ECM constituents, and integrins cooperate to enable growth factors to induce keratinocyte proliferation. Growth factors that MMPs may break down and convert into active forms include IGF-1. Furthermore, they remove the growth hormones from the wound matrix by using their proteolytic capacity. Growth factor receptor signaling pathways may be altered by the ECM's propensity to bind with integrins, leading to increased growth factor activity [58]. Growth factors, the ECM, and integrins interact to regulate keratinocyte proliferation during re-epithelialization of the epithelium. Activated keratinocytes that were recently deactivated ultimately resume their normal differentiation pathway to heal damage. When a layer of cells from the epithelium without discharge completely covers the lesion and is surrounded by a layer of keratinocytes, layering begins and signals that promote development cease [58]. Keratinocyte proliferation and migration in chronic wounds keratinocytes in nonhealing wound edges or intact epidermis are different from those in chronic wound edges in terms of morphology and physiology. Keratinocytes are restricted to the basal layers on healthy skin, however, in chronic wounds, they proliferate into the top layers. C-myc is activated and overemphasized, which results in the hyperproliferative epidermis [59]. Another possible explanation for the distinct structure of the epidermis is the instability of the activation and differentiation pathways. Filaggrin and K1/K10 proteins [59], as well as a particular group of small proline-rich proteins, were shown to be less abundant in venous ulcers. Involucrin and transglutaminase 1 are illustrations of late-stage differentiation markers that increased instead. Chronic nonhealing wounds have epithelium that expresses the precursor of the second chain of LN5 [60] less often, preventing the migration of these cells. Furthermore, keratinocyte nuclei and the cornified layer of chronic wounds may contain catenin. When these components come together, the epidermis experiences excessive cell division instead of migration. This epidermis cannot repair the protective barrier [59, 61].

## 6 FIBROBLAST GROWTH FACTOR (FGF)

In brief, tissue engineering has been identified as a potential method of repairing destroyed or sickly tissues in recent decades, and it has garnered significant funding for investigation [62]. Growth factors play an essential function in tissue creation by providing chemical messengers to stem cells that regulate their biological processes and tissue specialization. Though their fundamental biological functions and their innate contribution to tissue formation and repair remain less thoroughly investigated, growth variables recently caught the attention of many researchers when it comes to their use in tissue engineering approaches. Their use as a targeted agent to influence certain tissue responses is therefore feasible. Research has shown that FGF, a well-known growth factor, may have an impact on the regeneration and repair of tissue [63]. There are now 22 members in this protein family, and it was shown that the protein might stimulate the proliferation of fibroblasts. Many of the effects of FGFs are mediated *via* FGFRs. The primary signaling cascade triggered by the treatment of FGFRs is the RAS/MAP kinase pathway. Many damaged tissues, including skin, blood vessels, muscle, fat, tendon/ligament, cartilage, bone, tooth, and nerve, have been repaired using FGFs because of their potential biological activity. Next, the expected supply of FGF for tissue repair is used in addition to the transgenic human FGF family. In some previous experiments, FGFs, like additional growth variables, were given topically to the wound sites (Fig. **6**).

### 6.1 FGF Biology

The brain and pituitary gland are the primary destinations of acidic FGF (FGF1) and fundamental FGF (FGF2), which are fibroblast growth factors. Since then, at least 22 different FGFs have been found or divided. Invertebrates and insects have been reported to contain FGFs. Numerous vertebrate species have been shown to possess multiple FGF genes. For example, the FGF genes of zebrafish are ten (FGF2-4, 6, 8, 10, 17a, 17b, 18, 24), the FGF genes of Xenopus are six (FGF2-4, 8-10), the FGF genes of chickens are thirteen (FGF1-4, 8-10, 12, 13, 16, 18-20), and the FGF genes of mice and humans are twenty-two (FGF1-18, 20-23) and sixteen (14), respectively. Twenty-two different proteins make up the human FGF family: FGF1, FGF2, FGF3 (INT2), FGF4, FGF5, FGF6, FGF7 (KGF), FGF8 (AIGF), FGF9, FGF10, FGF11, FGF12, FGF13, FGF14, FGF16, FGF17, FGF18, FGF19, FGF20, FGF21, FGF22, and FGF23. There are only 18 ligands for FGFR, comparable to the 23 members of the FGF family. Although belonging to the same family as FGF, FGF11, FGF12, FGF13, and FGF14 do not interact with FGFR in the precise same manner as FGF homolog factors. Thus, categorizing them as FGF homologous elements is a more precise classification. It is also crucial to remember that the human FGF15 gene does not exist. Rather, the gene known as FGF19 [64] is comparable to the mouse FGF15 gene. The human FGF gene family is divided into seven subfamilies: FGF1, FGF4, FGF7, FGF8, FGF9, FGF11, and FGF19, according to phylogenetic analysis. The FGF1, FGF4, FGF7, FGF8, FGF9, FGF11, FGF11, 12, 13, and 14 and FGF19 subfamilies are composed of FGF1 and 2, FGF4, 5, and 6, FGF3, 7, 10, and 22, FGF8, 17, and 18, FGF9, 16, and 20 and FGF11, 12, 13, and 14 correspondingly. Six subfamilies of the human FGF gene family have been identified by gene location analysis: FGF1/2/5, FGF3/4/6/19/21/23, FGF7/10/22, FGF8/17/18, FGF9/16/20, and FGF11/12/13/14 [65].

### 6.2 FGF Signalling

FGFs work as molecules of signaling by adhering to and triggering FGFRs. After activation, particular molecules are drawn to the receptors cytoplasmic by modified tyrosine, which then binds to the receptor to initiate signaling. The process starts an assortment of signaling pathways that cause numerous responses in biology. These points are occupied by the SH2 (Src homology-2) or PTB districts of signaling enzymes, adaptors, and docking proteins. The creation of signaling complexes that are drawn to active receptors results in phosphorylation processes [66]. The AKT/PI3 kinase pathway, the PLC pathway, and the RAS/MAP kinase pathway have all been studied in the greatest detail. The PLC route, the PI3 kinase/AKT pathway, and the RAS/MAP kinase pathway are the three pathways that the FGF signal diagrammatically depicts. The biological functions of FGF bond to high-affinity tyrosine kinase FGFRs on the target cell's outermost layers and carry out physiological tasks, as previously explained. Thus, the FGF signal channel that links FGFRs and the FGF family is essential to FGF. FGFs play roles in angiogenesis, migration, differentiation, and cell proliferation in a broad spectrum of organs and tissue, as shown by numerous studies. It has been shown that FGFs stimulate the proliferation of several cell types, such as epithelial, endothelial, and embryonic stem cells. FGF1 is a growth-promoting hormone that may affect how fat cells develop in the human body. Furthermore, when FGF1 is given together with FGF2 and FGF7, the growth rates of IEC-6, Caco-2, and HT-29 cell lines accelerate [67]. When FGF2 arrives, the targeted exchange of genes between the telencephalon and mesencephalon of E10 mice produces neuroepithelial cells, which are more viable and grow more rapidly. Because trophoblast advancement necessitates FGF4, mice removing FGF4 embryos fail to survive after transplantation [68]. The rapid growth of cells in the epidermis has been associated with FGF7, commonly referred to as human KGF. Because FGF10 stimulates the growth of epithelial cells, prostate cancer progresses more quickly. Cell migration is the formation and survival of organisms with many cells depending on the migration of cells. The coordinated migration of cells over predetermined routes to specific locations is necessary for immune system responses, wound healing, and the growth of tissues throughout early life [69]. Cells often exhibit chemotaxis, their capacity to move in the interaction to and in the direction of certain external signals. Distinct migration of cell patterns is displayed by separate FGF subfamilies. In mice, FGF1 and FGF2 appear to have a significant effect on cochlear ganglion neuronal migration [70].

#### 6.2.1 Cell Differentiation

The term “cell differentiation” in developmental biology refers to a process wherein a less differentiated kind of cell gives way to a more specialized type of cell. Multicellular organisms go through several changes throughout their life cycle, from a single zygote to a huge network of tissues and cell types. In tissue repair and steady cell turnover, adult stem cells proliferate to become highly specialized replicating cells. The differentiation process significantly alters a cell's size, shape, membrane potential, metabolic activity, and sensitivity to external stimuli. The FGF subfamilies exhibit differences in cell differentiation. FGF1 and FGF2 exert significant effects on the mice's cochlear ganglion neuron early growth [50].

#### 6.2.2 Angiogenesis

Angiogenesis is a mechanism in which fresh arteries originate from existing vessels. It needs to occur for an array of physiological and medical conditions, including tumor growth, wound repair, inflammatory processes, and embryonic growth [71]. Stimulated endothelial cells burst out of the basement membrane to initiate the complex process of angiogenesis. Subsequent migration and multiplication of these tiny cells culminate in the development of solid endothelial cells sprouting in the stromal location. After the formation of tight connections and the deposition of a new underlying membrane, vascular loops and capillary tubes become prominent [72]. Several, including members of the VEGF family, angiopoietins, TGF-alpha and beta, PDGF, TNF-alpha, interleukins, chemokines, and FGF members, have been realized as inducers of angiogenesis [73]. However, studies on the angiogenic capacity of the 22 members of the FGF family in both *in vitro* (lab) and *in vivo* (living) species have merely glanced at a tiny portion of these properties. FGF1 and FGF2 have been demonstrated to have angiogenic functions. Endothelial cells are organized into structures resembling tubes with the assistance of FGF1 and FGF2, which also stimulate their growth. Thus, they encourage the formation of new blood vessels or angiogenesis. The angiogenic activity of FGF1 and FGF2 is stronger than that of VEGF or PDGF.

## 7 INSULIN-LIKE GROWTH FACTOR (IGF)

The insulin-like growth factor (IGF) system is composed of the hormone insulin, IGF-1, and IGF-2 peptide ligands, and both together. The external ligands demonstrate different levels of tolerance when it comes to activating the insulin receptor (INSR), IGF Type 1 receptor (IGF-1R), and IGF-2. IGFBPs influence the control of the extracellular system [74]. Additionally, it has been shown that ligands and protein binders interact with IGF family receptors, with their effects differing according to the type of cell and tissue. IGF-1 played a well-acknowledged role in assisting the growth and maturation of sound tissues [75]. In the endothelium, stroma, and epithelium of the cornea, the expression of INSR, IGF-2R, and IGF-1R together with their ligands has been investigated. The IGF family has significant biological functions in wound healing, internal stability maintenance, cell specialization, and cell proliferation, based on these findings and observations in other tissues (Fig. **7**).

A significant number of cellular proteins, such as those belonging to the families Shc, Rac, Ras/Raf, and IRS1/IRS2, are autophosphorylated by the tyrosine kinase IGF1R. Activated to regulate Rac and Ras/Raf in response to phosphorylation, the Rac pathway facilitates cell migration, whereas the Ras/Raf path is essential for proliferative reactions. Whereas IRSs interact with signaling molecules, activating the PI3K and Akt pathways concurrently initiating protein synthesis and avoiding death. Characterization of IGF-1R, IGF-2R, AND INSR in the human cornea isoform performs unique roles in metabolism and development. Each expression modulates kinase activity and insulin affinity, and it may also have an impact on the pace of internalization and receptor recycling [76]. INSR in the cornea was initially discovered by Naeser in the late 1990s [77]. The study used immunohistochemical methods to stain human corneas from donors with and without diabetic retinopathy for INSR. The staining procedure was unaffected by diabetes. To reveal the occurrence of INSR in the corneal epithelium, Rocha and collaborators used immunohistochemistry [78]. Moreover, they found that IGF-1R translocation into the cell's nucleus was assisted by SUMOylation or the attachment of the SUMO modifier SUMO-1 to IGF-1R [79]. More recent research has shown the control function that nuclear IGF1R plays in a range of nuclear proteins, including those involved in invasion, metastasis, DNA damage responses, and the cell cycle [80]. They also showed that adding trypsin to split nuclei affected the process of insulin binding. The scientists revealed that rat liver nuclei have an insulin-regulated hormonal receptor. Ten years later, some other set of individuals refuted these previous conclusions. Through an assortment of *in vitro* approaches, Soler *et al.* investigated the potential of a nuclear-localized receptor [81]. Unlike previous studies, they discovered that the hormone did not lose its physiological properties and continued to be active even after it separated from INSR, identified and linked to heterochromatin in the nucleus. Rat hepatocyte nuclei have shown the ability to receive INSR, which regulates dividing cells and calcium signaling. Hancock *et al.* looked at INSR's role in the mouse hepatocyte nuclear partition [82]. Through its interactions with RNA polymerase II, the scientists found that INSR communicates directly with promoters across the genome to control gene expression. Our findings support earlier studies by demonstrating the presence of INSR and IGF-1R in corneal epithelium cells' nuclei. Our findings are distinct from those of other studies as we have found that Hybrid-R [83] is the receptor with nuclear localization. Additionally, we discovered that the absence of growth factors essentially drives Hybrid-R's translocation to the nucleus rather than IGF-1. Insulin, however, controls the placement and function of every receptor [84]. IGF1R and INSR expression are elevated in low-insulin environments, leading to the accumulation of these receptors as Hybrid-R in the nucleus of corneal epithelial cells. The SUMOylation mechanism, known for mediating this function, depends on the SUMO2/3 modifier. Insulin is a peptide hormone that regulates growth, proliferation, and metabolism. Disulfide bonds join the A- and B-chains, combining constitute the fundamental components of human bioactive insulin. It is found as a monomer. Insulin is produced and retained by beta cells in the pancreas, and as soon as blood glucose levels spike, insulin is released [85]. Insulin has a vital function as it promotes glucose absorption in the majority of cells and tissues. Insulin increases the absorption of glucose from the blood into various tissues, including the lungs, adipose tissue, and skeletal muscle, by binding to the insulin receptor (INSR) and activating the (GLUT4). Nonetheless, certain tissues, including the corneal epithelium, absorb glucose without the need for insulin [86]. The enzyme that facilitates the digestion of glucose is the glucose transporter GLUT1, which is always active. Consequently, glucose may enter cells continually. When there is an increase in energy needs, the corneal epithelium reacts by expressing more GLUT1 transporters, much as in a wound. This adaptive response ensures that there is enough amount of energy available for survival, migration, and proliferation [87]. Clinically, these alterations show up as chronic abnormalities in the epithelium, treatment-resistant corneal wounds in diabetic patients, altered function of the epithelial barrier, recurring epithelium erosions caused by an aberrant basement membrane, and superficial punctate keratitis [88]. The absence of corneal nerves results in modifications to cells and an overall reduction in corneal sensibility, which diminishes the corneal layer and lowers tear production. The latter is often the cause of dry eyes [89]. The etiology of corneal disorders can be complex and depends on the build-up of reactive oxygen compounds as well as dysregulated growth factor signaling [89]. It has been suggested that applying insulin straight to the affected region could accelerate the healing process of corneal epithelial lesions. This is due to insulin's ability to accelerate cell growth in laboratory conditions. Zagon *et al.* used STZ to persuade diabetes in Sprague-Dawley rats for their research [90]. The investigators constructed a 5 mm corneal incision and then delivered topical insulin on four occasions a day for seven days. Contrasting topical insulin treatment to the control material, it showed an increase in both the bottom layer's new cell emergence and the outermost layer's skin cell regeneration. The resumption of normal corneal sensitivity was a significant result of offering insulin to the impaired corneal area. It also indicates that insulin influenced the method of corneal re-innervation. The structure of the peptides IGF-1 and IGF-2 are shared, and they affect how body cells grow and concentrate. Activation of their cognate receptors causes autophosphorylation of their intracellular kinase domain and causes the processes of PI3K, MAPK, and JAK/STAT [91]. IGF-1 has garnered significant interest due to its vital role in the movement of cells and their proliferation in tissues other than the retina. It has been shown that IGF is essential for wound healing, maintaining homeostasis, and controlling the three cell types of the cornea's activity. In the corneal epithelium, IGF-1 promotes cell proliferation. Hybrid-R activation is the first step in the chain of events that contributes to the phosphorylation of Akt [92]. It has been shown by Lee and colleagues that IGF-1 stimulates the migration of corneal epithelial cells in addition to β1-integrin and Lamin-5 expression. The findings clearly show that the differentiation of limbal stem cells into corneal epithelial cells requires IGF-1. Furthermore, there is increased expression of IGF-1R in the limbus. Increased IGF-1R expression in the limbus, which has, in turn, promoted superior limbal epithelial cell development, is shown by elevated cytokeratin K12 levels. Analogous conclusions on the influence of IGF-2 were derived from further research using a similar methodology [93]. IGF-1 not only affects the corneal epithelium but also the corneal stroma, promoting regrowth there as well. Corneal keratocytes, the owner cells in the stroma, convey two key roles both preserve the stroma's structural integrity and offer a cogent network of communication throughout the cornea [94]. IGF-1 is vital for the keratocyte maturation cycle. Stentormal cells adapt to harm and inflammation by activating and completing the differentiation process. IGF-1 is produced when HCE and primary cultured corneal fibroblasts [95]. Research demonstrates that IGF-2 is involved in the development of the mouse eye [96]. IGF-2's role in the postnatal development of the cornea remains to be investigated. To do this study, Park *et al.* utilized keratocytes that were gathered from the corneas of rabbits and cows. The scientists looked at collagen synthesis as well as the release of IGF-2 and IGFBP-2. Substantial variations have been identified. In a controlled environment, rabbit keratocytes were cultured to produce type I collagen and IGF-2. On the other hand, the release of IGFBP-2 by bovine keratocytes has been reported instead of IGF-2. IGF-2 could play a key role in collagen synthesis since bovine keratocytes grown in rabbit keratocyte-conditioned medium revealed enhanced proliferation and collagen deposition. Corneal fibroblasts cultured on plastic with increased IGFBP-2 levels have been found to secrete more keratocan and aldehyde dehydrogenase (ALDH1A1) [97]. Inactive indicators of keratocytes. Keratocytes were raised on plastic cells and stimulated with TGF; the resulting myofibroblasts were characterized by a small amount of IGFBP-2 and elevated amounts of smooth muscle actin (-SMA) [98].

## 8 BASIC FIBROBLAST GROWTH FACTOR (BFGF/FGF-2)

Ovarian corpus luteum, a temporary endocrine gland, arises from the surviving cells of the predominant follicle after fertilization [99]. FGF2, additionally referred to as basic fibroblast growth factor, and VEGFA are powerful chemicals that promote endothelial cell division. They are also in charge of luteal tissue proliferation, an indication of rapid angiogenesis. Inhibition of FGF2 activity during ovulation resulted in lower blood levels of progesterone and dysfunctional corpora lutea in cow studies [100]. Luteinizing progesterone is a key hormone that helps prepare and maintain the uterine lining, which promotes the emergence and maintenance of a normal pregnancy. In no evidence of pregnancy, the corpus luteum promptly regresses both physically and functionally, resulting in a decrease in the concentrations of progesterone and luteal tissue. The resulting fast regrowth of tissue contributes to the damaged mass known as the corpus albicans. The ruminants initiate this process with a hormone called prostaglandin F2 (PGF2), which originates in the reproductive system [100]. When FGF2, a growth factor that binds to heparin, is present, 3T3 fibroblasts may multiply. It exhibits pleiotropic effects in certain organs. The FGF signaling peptide family encompasses 18 secreted proteins that specifically interact with the four distinctive tyrosine kinase FGF receptors (FGFRs 1-4). The receptors that FGF2 predominantly interacts with are FGFRs-1c, -2c, and -3c. A factor referred to as “ovarian angiogenesis” promotes the growth of new blood vessels in the ovaries, and this is what FGF2 does. It has been shown that microvascular endothelial cells are cells of the corpus luteum that contain FGFRs at a specific site. Moreover, it has been shown that luteal steroidogenic cells express FGF2 in conjunction with FGFR [101]. FGF2 stimulates the AKT1, MAPK1/3 (ERK1/2), and STAT1 signaling pathways, which in turn promote transcription and subsequent cell division [101]. FGF2 encourages the luteinizing granulosa cells *in vitro* to produce more progesterone. However, the kind of cell culture and its duration could affect this result. Other research indicates that FGF2 has no effect on humans and cow progesterone-producing luteinizing follicular cells [102]. Lower doses of FGF2 have been seen to elevate progesterone release in luteal cells from sheep and bubaline, resulting in greater stability of outcomes in primary luteal cells. Previous studies have shown increased progesterone release after FGF2 injection into microdialysis bovine corpora lutea [103]. Fibroblasts are cells with mesenchymal characteristics that produce and maintain the extracellular matrix. These cells express actin (ACTA2), also known as alpha-smooth muscle actin (SMA), while being stimulated to develop into myofibroblasts. While fibroblasts have been shown to exist in the corpus luteum, the number of SMA-positive cells in bovine follicles was raised by FGF2 treatment. It follows that FGF2 controls the fate of the corpus luteum. It is important to point out that TGFB1 enhances early luteal regression and is believed to have a crucial role in triggering fibrotic responses in several organs. In a previous investigation, it was shown that TGFB1 failed to promote luteal fibroblast growth. Furthermore, the introduction of chemicals known to induce death in steroidogenic and endothelial cells did not diminish the life of luteal fibroblasts. This research indicates that fibroblasts are the sole living cells in luteal tissue that endure luteal regression. FGF2 was shown to be higher in corpora lutea that were in the late stage and exhibiting spontaneous regression. When treated with FGF2, fibroblasts originated in a number of organ systems exhibited increased migration, proliferation, and VEGFA production. Furthermore, it has been shown that fibroblasts in the flexor tendons and heart need FGF2 to help them create an extracellular matrix. Comprehending the functions of FGF2 in the formation of collagen and luteal regression can help with an explanation of how chemicals generated in the first phases of luteal regression influence tissue remodeling and the later development of corpus albicans. The intent of our study sought to examine the theory that FGF2 promotes the proliferation of bovine luteal fibroblasts and boosts their capacity to produce collagen. Numerous signaling pathways, including ERK, AKT, and STAT1, have shown functional effects upon exposure to FGF2 (Fig. **8**) [104].

Cell proliferation measurement live cell counts and EdU incorporation, 8.4 × 10^4^ cells per well on a 6-well dish (at 50% confluence) were used, whilst 4.2 × 10^4^ cells per well on a 12-well plate were employed for western blotting. These densities were used to assess the generation of collagen and the proliferation of cells. The trypan blue exclusion technique was utilized to determine the number of live cells to assess proliferation. Bovine luteal fibroblasts on plates were organized according to earlier instructions. The cells were allowed a half-hour on a subsequent day to acclimatize before the medium was replaced with fresh high-glucose DMEM that included 10% FBS. Following acclimation, a concentration range of 0 to 10 ng/mL of FGF2 was administered. The duration of the incubators was either 24 or 48 hours. To get the cell count, the cells were split using TRYPLE solution, washed with 1XPBS, and quantified using a Countess Automated Cell Counter (ThermoFisher Scientific, Waltham, MA, USA). A proliferation experiment utilizing the click EdU Andy Fluor 647 Imaging Kit (ABP Biosciences, catalog # A006; Beltsville, MD, USA) revealed the effect of FGF2 on cell growth. EdU, or 5-ethynyl-2′-deoxyuridine, was one of the experiment's components. As mentioned before, luteal fibroblasts were set up on a plate. The cells were washed and cultivated in DMEM supplemented with or without 10% FBS after 24 hours. After the adaptation interval, half of the media was replaced with a medium containing 10 M EdU and 5% FBS. The fibroblasts were then treated with either a control medium or FGF2 (1 ng/mL) for 48 hours. The wavelength at which EdU-containing nuclei were found was 647 nm, whereas the remaining nuclei were identified at 405 nm using Hoechst. The quantity of both total and EdU-positive nuclei was assessed by calculating the ratio of nuclei to the control, and the cell counter tool in ImageJ was used to assess the results. For each slide, ten photos were taken at a magnification of 10 (NA = 0.30). When all 10 images are merged, they show an average of one biological replication. FGF2 promotes the development and proliferation of luteal fibroblasts. We explored the topic to discover how FGF2 regulates the development of bovine luteal fibroblasts. Specifically, we explored the protein expression of the PCNA, which is a recognized marker for cell proliferation. In addition, the number of living cells was established and their incorporation of the DNA synthesis marker EdU was assessed. After luteal fibroblasts were treated with FGF2 for a whole day, there was a significant rise in PCNA content (n = 3), but not in cell number (n = 3). Despite this, the PCNA found in bovine luteal fibroblasts increased after the 48-hour injection of FGF2, leading to a faster rate of proliferation at dosages of 1 and 3 ng/mL (n = 5, *p* < 0.01). We assessed the incorporation of EdU into bovine luteal fibroblasts to evaluate cellular proliferation. For that reason, serum-free or media containing 5% fetal bovine serum (FBS) was utilized to culture bovine luteal fibroblasts for 48 hours. FGF2 was incorporated either way, either at a concentration of 1 nanogram per milliliter. An evaluation of EdU's DNA integration came next. EdU absorption increased 1.5 times in serum-free conditions (*p* = 0.056), but it enhanced 1.7 times in 5% FBS medium (*p* = 0.051) [105]. To identify the signaling pathways necessary for the proliferation of luteal fibroblasts stimulated by FGF2, tiny molecule inhibitors were used to hinder the roles of the upstream mediators that had previously been discovered. The luteal fibroblasts were pre-incubated for one hour preceding being injected with FGF2 for an interval of 24 to 72 hours. The present study used a sample size of five individuals. Proliferation was determined by cell count enumeration and PCNA by western blot analysis. After a brief (5-30 min) treatment with FGF2 at a concentration of 1 ng/mL, a western blot analysis was performed to confirm the inhibitor's effectiveness before the experiment. Using the Western blot analyses, it was feasible to see that the AKT kinase inhibitor effectively decreased AKT activity while the MEK inhibitor U0126 successfully reduced ERK phosphorylation. At 48 and 72 hours, FGF2 was able to momentarily increase PCNA levels, even though the MEK inhibitor was able to reduce basal and FGF-induced ERK signaling and lower baseline PCNA levels. Additionally, ERK inhibition was associated with a 0.79-fold decrease in the number of cells activated by FGF2 (*p* < 0.05). AKT inhibition, on the other hand, significantly reduced FGF2's ability to raise PCNA levels (*p* 0.0001) and cell proliferation (*p* 0.001). FGF2 stimulates the synthesis of collagen in luteal fibroblasts. We conducted three trials (n=3) to examine the effect of FGF2 on collagen synthesis since fibroblasts are crucial to the process. When serum was not present, FGF2 substantially raised the quantity of type I collagen. This spike was at least 1.8 times higher at quantities > 0.3 μg/ml after 48 hours of medication (*p* < 0.05). We illustrate, for instance, that luteal fibroblasts generate much more collagen with the administration of TGFB1 (*p*<0.001) [104].

## 9 NERVE GROWTH FACTOR (NGF)

The process of healing a wound involves numerous aspects and requires several stages to complete. Acute inflammation is the first response to an injury, and then mesenchymal and parenchymal cells migrate, become activated, and proliferate. Another substance that forms and deposits is the extracellular matrix. Inflammation, tissue formation, and tissue remodeling are the three stages in succession that constitute the wound-healing process and are controlled by sophisticated cytokines networks [106]. In rodents, eliminating the submandibular gland slows down the healing of wounds induced during experimentation, according to previous research. Mice's saliva contains significant amounts of NGF, which the submandibular glands generate in enormous quantities. Research has shown that the insertion of NGF topically in rats with removed salivary glands has a substantial impact on the rate of wound healing [32]. This indicates that, in addition to other contaminants that promote healing, such as FGF and EGF, licking injuries could release NGF. Thus, NGF in saliva likely serves a physiological role by promoting the healing of wounds once a person licks a wound. In addition to its well-known role as a neurotrophic factor, recent research suggests that NGF has biological effects on nonneuronal cells, including inflammatory cells (mast cells, neutrophils, and macrophages) and other cell types (keratinocytes, fibroblasts, and endothelial cells) that are crucial for wound healing. The focus of the present review is to investigate the role and methodology of NGF in tissue repair and wound healing, as shown by animal studies. It also aims to highlight the potential advantages of using NGF as a medication to treat a range of illnesses. After surgery, clean incisions go through a series of steps, as previously explained, to aid in their healing. The wound heals swiftly behind a temporary fibrin clot barrier. Within twenty-four hours, the growth of neutrophils peaks at the injury site, and within forty-eight hours, epithelial cells start to migrate along the edges of the incision. On day three, when the amount of cell granulation and monocytes in the affected area reaches its maximum, collagen fibers begin to develop. After the first week, up to seven days have elapsed, and collagen begins to develop over the injury. During the generation of the epidermis' function, its dermal structures sustain permanent damage. Over a week, the injury has only regenerated to 10% of its initial state. But after just two or three months, the wound's resilience has risen significantly, achieving a range of 70%-80%. How rapidly a lesion heals depends on several characteristics, notably the site, type of tissue, bacterial status, glucocorticoid usage, and circulation disruption [107].

### 9.1 Cellular Events during the Wound Healing Process

The process by which dangerous germs are eliminated from wounds and tissues that are damaged are either entirely or partially regenerated can be referred to as wound healing. Three fundamental phases can be distinguished in the wound healing process, which are interrelated and overlap: inflammation, tissue creation (granulation, angiogenesis, and reepithelization), and tissue remodeling (Fig. **9**) [107].

Inflammation coagulation, a fast reaction that accumulates circulating platelets and forms a clot in a couple of minutes, serves as a preventive strategy against excessive blood loss, as most traumas cause damage to the vessel walls. VEGF, PDGF-BB, and TGF-β are among the growth factors released by activated platelets. Vasodilation and blood vessel susceptibility are facilitated by these growth factors. Additionally, they cause inflammatory cells to go to the injury location [108]. Tissue development moves to the wound site; endothelial cells, cells of the epidermis, and fibroblasts proliferate in that area. A variety of factors, such as the release of cytokines and growth factors by activated macrophages and the disruption of cell-to-cell contact, influence the motion of cells in residential areas. A few hours later, epithelial cells that have started to move and develop occupy the visible region. Re-epithelialization is an important stage in the recuperation of surgical wounds, given that it creates an interim wall and facilitates wound contraction. The abundance of epithelial cells near the wound's border increases the expression of matrix MMP family members. MMPs contribute to the movement of cells in two distinct capacities both disintegrate elements of the matrix and function as regulating molecules. To achieve this, they enzymatically convert inactive forms of matrix, adhesion molecules, and cytokines into physiologically active forms. Tissue remodeling is a normal process of repairing an injury, components of connective tissue involving collagen and proteoglycans are synth broken down quickly [109, 110]. Compared to earlier wounds, one-week-old wounds have more collagen. Collagen rebuilding, however, increases the tensile strength of the second wound. MMPs and serine proteases are released by keratinocytes, damaged epithelial cells, and neutrophils. They control how these structural parts break down through the cytokine network. A protein known as the tissue inhibitor of MMP (TIMP), which blocks MMP's biological actions, is essential to the remodeling and wound-healing processes.

### 9.2 The Wound and Disease Healing Capabilities of NGF

#### 9.2.1 Peripheral Nerve Wound Healing

The biology of nerve injury and regeneration is based on a complex framework comprising the relationships of the nerve cell's body, axonal fiber, and its immediate milieu [110]. Peripheral nerve cells in the mature PNS can impressively survive axonal destruction and even create new ones. Neurotrophins and their receptors are overexpressed, and there are more neural cell adhesion proteins and other cytokines in the distal stump. This causes changes in the cells. The findings of Levi-Montalcini and Hamburger in 1953 demonstrate that the neurotrophic factor, or NGF, enhances the neural development and survival of basal forebrain cholinergic neurons and peripheral sensory neurons. Target cells' susceptibility to NGF can be assessed by the existence of two unique outermost cell receptor types: the low-affinity neurotrophins receptor (p75NTR) and the high-affinity TrkA receptor [111].

#### 9.2.2 Skin Wound Healing

Studies have shown that NGF, a neurotrophic factor necessary for the development and function of neurons in the peripheral and central nervous systems, influences the immune and inflammatory responses in nonneuronal tissues. Increased NGF levels in the epidermis are associated with skin innervation [112]. Skin is home to NGF and its receptors. These findings suggest that neurotrophins, including NGF, have a significant impact on skin biology. Scientists have examined other functions of NGF in inflammation and tissue regrowth. Similar to this case, NGF promotes granulocyte development in hematopoietic stem cells from humans and mice [113]. NGF enhances the activity of some inflammatory cells, including neutrophils, macrophages, and monocytes. It also prevents mast cell and neutrophil mortality. NGF is generated naturally by a wide range of epidermal cell types, which include fibroblasts, mast cells, and keratinocytes, suggesting that NGF may be involved in the healing of cutaneous wounds [114]. Recent research has shown that topically administering NGF accelerates the healing of skin lesions in rats. NGF cannot be detected in the blood before full-thickness skin-punched wounds, although serum NGF levels rise swiftly after the wounds. On day 14, there was an extended recovery to the baseline NGF level of 7.8 ng/g wet volume one day after the injury. The area of the epidermis that suffered harm had a much greater concentration of NGF than the part that was not. A sialoadenectomy significantly decreased the rise in blood NGF levels before wounds; however, the skin lesion was not similarly impacted. By this, NGF is released into the bloodstream very soon after salivary gland injury. The *in-situ* hybridization technique was employed to determine precisely the cell type that induces NGF in the damaged skin. Granulation tissue, fibroblasts, and epithelial cells were all exhibiting mRNA-NGF signal expression at the wound site [114].

### 9.3 Healing of Wounds in Tissues Other than Skin

#### 9.3.1 The Organ Responsible for Vision

NGF is also associated with a potent visual stimulant that facilitates the healing of wounds [115]. The quantities of mRNA NGF and mRNA TrkA were measured in rabbit retinal tissues that had undergone both experimental and normal iridectomies. The lens, iris, cornea, and cilia all contain NGF and TrkA. NGF levels vary from 8.93-3.97 ng/g in the iris, representing the most elevated level, to 0.023-0.001 ng/g in the aqueous humor. After iridectomy, NGF levels in the iris, ciliary body, and aqueous humor improve substantially promptly *versus* patients who underwent an imaginary surgery. Conversely, there is no corresponding increase in the cornea or lens. In rabbits, therapeutic ocular hypertension induces harm to the retina and enhances local NGF production. Rats' and competent people's corneas were additionally found to have NGF [116]. Human and rat corneal epithelial cells produce, store, and release NGF in a lab context. They additionally express TrkA. Rats with corneal epithelial lesions temporarily elevate nerve growth factor (NGF) levels inside their corneas. Neutralizing anti-NGF antibodies stops the body from making its own NGF, which makes retinal damage worse and speeds up the healing of corneal epithelial cells. However, the application of NGF topically quickly speeds up the recovery process. Whenever bone suffers harm, its ability to regenerate is amazing. As previously mentioned, we can divide the complex process of healing a fracture into three distinct stages: remodeling, tissue creation, and inflammation. During the tissue regeneration phase, osteoblasts, specialized cells responsible for bone formation, solidify a cartilage-like bone matrix, forming a callus. The last stage involves the remodeling of the bone by deposition and resorption. An animal model was previously used to study the role of NGF in bone development after a synthetic bone fracture [117]. Although NGF and TrkA are constantly produced in bone tissues, the amount made increases during the fracture healing process [118]. The main goal of autotherapy is to treat muscular injuries. Muscle cells can respond to NGF, while TrkA expression is frequently observed in both human and murine myoblast cell lines [119]. NGF promotes the proliferation of slowly dividing primary myoblasts and speeds up the fusion of core myoblast cells and cell lines. In a mouse model of muscle contusion, the genesis of muscular fibrosis correlates with an event of substantial muscle regeneration occurring over two weeks. By stimulating myoblast differentiation and proliferation, NGF treatment to damaged areas promotes muscle repair. However, the study team found that in the setting of muscle damage wound repair, NGF is less beneficial than IGF or FGF [120]. The amount of NGF administered determines tooth growth. Studies have shown that in a research environment, rat tooth pulpal cells encourage the proliferation of nerve fibers from trigeminal neurons [121], revealing that these cells release growth-promoting substances such as NGF. Researchers found that NGF and TrkA receptors are present in the gingival fibroblasts, keratinocytes, and dental ligament cells. When there are lesions in the tooth's pulp, sensory nerve fibers often proliferate significantly, and fibroblasts in the pulp of the tooth produce more local NGF (nerve growth factor). Due to the location of NGF and its receptor, NGF not only influences tooth innervation but also plays nonneuronal, organogenetic roles during tooth development and repair. Currently, there is a dearth of accessible data [122].

## 10 HEPATOCYTE GROWTH FACTOR (HGF)

Adult bone marrow-derived MSCs possess a remarkable capacity to differentiate, which allows them to differentiate into cell lineages distinct from their original tissue [123]. This capacity for adaptation may be employed by the body to reclaim tissue’s structure, coordinate cell regeneration, and substitute cells that are damaged. This aptitude might be shown *via* unknown procedures and conditions. While it presents a major obstacle, identifying growth factors that specifically stimulate the movement, differentiation, and amplification of MSCs holds great promise for the advancement of stem cell-driven tissue engineering [124]. Endothelium and epithelial cells constitute some of a variety of cells that get assistance in their migration, proliferation, assault, growth, and survival by the multifunctional cytokine termed HGF secreted by mesenchymal cells. Both the production of organs during embryonic development and the maintenance of stable circumstances in adult tissues depend on the simultaneous integration of these activities. In the mammary and renal glands, HGF promotes the formation of blood vessels and tubule expansion. Moreover, it promotes hepatocyte survival and serves as a chemical trigger that recruits motor neuron axons and myoblast precursors. Adults' kidney and liver regeneration process is aided by HGF. It is cytoprotective to a variety of cell types that are present in seriously injured living beings' tissues. When the HGF signaling pathway is overactivated, cancer arises and tumor cells disseminate to other parts of the body. The physiologic processes that HGF induces are mediated by the met proto-oncogene-encoded tyrosine kinase, a high-affinity receptor for HGF [125]. The Met receptor glycoprotein is formed out of two different subunits that are bound together by disulfide bonds. Thanks to its kinase domain and specific docking place of residence, it may phosphorylate confident tyrosine residues and so form linkages to multiple signal transducers and adaptors, such as Gab-1, SHC, and GRB2 [125]. While the Met receptor has been linked to the ras-ERK1/2 MAPK pathway, Gab-1 has an extreme liking for PI3-kinase.

### 10.1 Proliferation Assay

Cell growth experiment, 96-well microplates held a total of 2.5 x 10^3^ cells. Before that, the cells were grown for a total of twelve hours in a medium that included 10% FCS. The cells were then starved of nutrients for a whole day by immersing them in a solution lacking 2% FCS. The cells were subsequently transferred to a 10% serum medium or a 2% serum medium-sized, with or without the addition of 20 ng of HGF per milliliter. New HGF medium plus and negative were incorporated, respectively, into the HGF medium plus and negative. In addition, the cells were treated with a solution that included 20 nanograms of hepatocyte growth factor (HGF) per milliliter, along with 10% fetal calf serum (FCS). The cells were exposed to 1 Curie per milliliter of [3 H]-thymidine, and they were then developed for 3 hours. Once trypsinization was completed, the cells were centrifuged and then subjected to 5% trichloroacetic acid (TCA) for thirty minutes at 4°C. Reconstructing the TCA-insoluble fraction included adding 0.1% SDS to 200 mM NaOH. After that, the samples were measured for radioactivity using a liquid scintillation counter (Packard Instruments, Tricarb 2180 TR). Numerous investigations have shown that cells were preincubated with certain inhibitors for an entire hour before stimulation. The student's t-test was implemented for statistical evaluation of the information [126].

#### 10.1.1 Evaluation of Transwell Migration

Transwell chambers from Corning Costar Italia in Concorezzo, Italy, were used for the chemotaxis tests. In brief, 200 liters of MSC-containing medium were positioned on the upper surface of a polycarbonate membrane with a porous structure, including 8 micrometer-sized pores. Five hundred microliters of medium with or without HGF with a 2% FCS content were delivered inside the bottom chamber. Throughout 48 hours, the plates were incubated at 37°C in a 5% atmospheric carbon dioxide environment. The K252A blocker was added to the cultures and incubated for one hour before starting the inhibition tests. The cells on the top side of the filter were mechanically removed after incubation. After the cells had migrated to the bottom side of the filter, they were immobilized for half an hour using an 11% glutaraldehyde solution. Particles were then stained with eosin and hematoxylin. Under every filter, 125 random fields were tallied, with five to ten fields per filter [126].

#### 10.1.2 HGF's Hepatotropic Functions during Liver Regeneration

One theory is that controlled cell growth occurs during regeneration of the liver. Rats with a 70% liver removal surgery had blood samples containing HGF, which was demonstrated to increase the growth of mature rat liver cells. During hepatic damage, it is seen that the two intra- and extra-hepatic activities cause a considerable increase in blood and liver HGF levels. When 70% of the liver's tissue has been eliminated, research employing c-Met gene eradication techniques or anti-HGF antibodies has shown that HGF's hormonal and local signaling activities contribute to the creation of the liver and the recovery from hepatitis [127]. This notion is shared by many different organs. We examine the mechanisms that control liver regeneration *via* a cascade of molecular events, with particular attention to the function of the HGF-c-Met system.

#### 10.1.3 HGF Plays Critical Functions in Liver Growth following 70% Hepatectomy

Rats having a 70% liver transplantation revealed an upsurge in HGF blood levels two distinct times before hepatocyte duplication. After a 70% hepatectomy, it was shown that intact distant organs such as the lungs, kidneys, and spleens had substantially higher levels of HGF mRNA levels than the enduring livers [128]. These are some of the first studies to indicate that the “endocrine” system plays a critical role in transporting HGF to the residual lobes *via* the vein known as the portal.

#### 10.1.4 The Role of the HGF-c-Met Pathway in the Process of Acute Hepatitis Repair

In hepatitis cases, the damaged hepatocytes are replaced by newly formed hepatocytes; this process of regrowth is called “*in-situ* repair.” To replicate the clinical situation of acute liver disease in humans, toxic chemicals such as CCl_4_ are used in investigations on animals. Using mice treated with CCl_4_ as a hepatitis model, we have collected evidence that indicates the paracrine system responsible for producing and spreading HGF is critical for the recovery from hepatitis. Rats with serious hepatitis show a more marked and early elevation in HGF mRNA levels in the liver than in other parts of the body, in contrast to a 70% hepatectomy [129]. Kupffer cells, referred to as sinusoidal cells, are the source of HGF during hepatitis, as shown by *in situ* hybridization [130]. Particularly, the administration of anti-HGF IgG after the CCl_4_ injection resulted in a decrease in the number of hepatocytes experiencing proliferation. The function of the HGF-c-Met axis in healing hepatitis may be replicated in mice whose livers have a particular deletion of the c-Met gene. According to this, the signal produced by surrounding cells like Kupffer cells serves as essential for the organ's ability to bounce back after hepatitis and restore its form and function (Fig. **10**).

Even if organs with damage have higher concentrations of systemic HGF, several organs may have HGF due to elevated plasma HGF concentrations. In other words, healthy organs tend to hinder blood HGF from stimulating c-Met, maybe because they do not need tissue repair. Exactly whatever chemical reaction is in charge of this occurrence is yet unknown. The serine component of the c-Met juxta region at position 985 (Ser-985) acts as a negative regulator, obstructing HGF-c-Met signaling, according to *in vitro* research. HGF does not induce epithelial dispersion when Ser-985 is phosphorylated. However, c-Met tyrosine phosphorylation is allowed and epithelium migration is stimulated when PP2A dephosphorylates Ser-985 [132]. Thus, a delicate balance between tyrosine phosphorylation and Ser-985 is a necessity for HGF-c Met signaling. Even in the presence of HGF, Ser-985 remains perpetually phosphorylation in non-injured tissues, prohibiting c-Met tyrosine phosphorylation. HGF supports epithelial regrowth in a range of organs, especially the kidney and lung, consistent with many *in vitro* tests [133]. HGF plasma levels were higher in patients who had suffered organ damage. Anti-HGF IgG therapy has been shown to disrupt the epithelial healing process in a variety of rodent organs. The results here demonstrate the way HGF promotes tissue regeneration in the kidney as well as other organs. Following a partial nephrectomy, the remaining kidneys develop in a manner that makes up for the loss of nephrons, much as the liver does. Though there are indications that blood-borne renotropic chemicals could be associated, the particular parts at fault are still unknown. Both intact organs, such as the lungs and remaining kidneys, have greater levels of HGF mRNA and protein before the start of renal regeneration. This suggests that paracrine and endocrine paths involving HGF have a consequence on kidney regeneration [134]. Exactly 24 hours later, mice experiencing acute lung injury demonstrated higher levels of HGF mRNA and protein. The lungs' bronchial and alveolar epithelium also reached the G1/S stage. Furthermore, when exogenous HGF was given to mice following lung injury, their alveoli and passageways revealed a more rapid rate of expanding cells [135]. Two studies that used mice as models demonstrated the involvement of endogenous HGF. The rats had temporary lung ischemia, which resulted in damage to their alveoli. However, after four days, the alveoli healed and the release of HGF increased. Particularly, anti-HGF IgG immunization blocked the alveolar growth of cells in a rat model of ischemic [136]. Throughout the breakdown and absorption of nutrients, the intestinal tract's mucosal membranes remain constantly under mechanical and chemical stress. To speed up the traumatized mucosa's healing process, daughter epithelial cells must be mobilized. External HGF medication aids in the recuperative phase of gastrointestinal injury. Anti-HGF IgG, on the other hand, prohibited the gastrointestinal mucosa's c-Met from being activated, which delayed the growth and migration of epithelial cells of the mucosal surface along the folds. The sanity of the stomach mucosa was subsequently damaged even after HGF was neutralized. In the mouse model of colitis, comparable findings were seen, with an increase in mucosa lining degradation and inhibition of the c-Met tyrosine phosphorylation process [137]. The local interaction between HGF and the c-Met receptor affects both tissue regeneration and maintaining metabolic homeostasis. HGF stimulates insulin synthesis and encourages the proliferation of pancreatic cells [138]. HGF is synthesized by pancreatic beta cells and influences insulin generation in these cells, hence assisting in the management of hyperglycemia. Notably, selectively removing c-Met from cells caused blood insulin levels to decrease, islets to shrink, and ultimately moderate hyperglycemia to develop in animals. The study suggests that the local HGF-c-Met circuit has a role in controlling blood glucose. HGF plays a critical role in the body's capacity to repair itself after injury to the muscles, cartilage, or skin. In a mouse study, anti-HGF (IgG) therapy was shown to block keratinocyte migration and proliferation, which are crucial cells involved in wound healing. Moreover, the HGF-c-Met signaling pathway aids in the growth and regeneration of skin-derived hair follicles [139, 140]. Myogenic cells generate HGF *via* the autocrine pathways in response to muscle damage. Anti-HGF IgG administration hindered myoblast multiplication and the formation of muscle fibers in animals that had suffered muscular injury. Chondrocyte formation is stimulated by HGF in controlled circumstances. It has also reportedly been shown to reduce cartillage [141] damage in rabbit knees [139]. Hematopoiesis likewise requires HGF. In a laboratory setting, HGF accelerates the growth and development of hematopoietic progenitor cell colonies, consistent with our observations. Elevated thrombopoietin exports in the hepatocytes might be the cause of raised levels of platelets in a group of HGF-transgenic mice. Notably, the livers of animals whose fetuses have c-Met deficiencies generate fewer blood cells overall [140].

### 10.1.5 HGF Production Sensing Systems during Liver Regeneration

The role of HGF in the liver's ability to regenerate after damage is becoming more well-recognised. Understanding how host cells generate and distribute HGF to wounded regions in line with the kind and severity of liver injury is thus essential. There are quite a few recognized drugs that are involved in the upregulation of HGF gene expression, disregarding the obstacles of finding agents that raise HGF in injured livers or plasma. Pro-inflammatory cytokines originate from resident or infiltrating macrophages in liver injury. Mediators that help prime hepatocytes for G0-to-G1 conversion and boost HGF synthesis include IL-1, IL-6, interferon, and TNF [131]. In simple terms, there is an expectation that inflammatory agents like TNF- and IL-6 play a part in liver regeneration. To get the liver cells ready for expansion, they first function as co-mitogens. Next, following hepatectomy, they produce more HGF, which is required for liver cells to migrate through G1 to the S phase (Fig. **11**).

## CONCLUSION

The review highlights the crucial roles of growth factors in the wound healing process across different phases. During the inflammation phase, growth factors such as PDGF and VEGF initiate the inflammatory response by attracting immune cells to the wound, a process essential for healing. In the proliferation phase, FGF and EGF promote cellular proliferation, migration, granulation tissue formation, and re-epithelialization, all of which are vital for restoring tissue integrity. In the maturation phase, TGF-β contributes to remodeling the extracellular matrix and scar formation, ensuring that the healed tissue regains functionality. Clinical implications suggest that understanding the roles of these growth factors can significantly enhance therapeutic strategies for various wound types, including chronic ulcers and surgical wounds. While some clinical trials have demonstrated potential benefits, further research is needed to develop standardized protocols for applying growth factors in clinical settings. To optimize the therapeutic use of growth factors, several actionable recommendations are proposed. These include targeted therapy, where specific growth factors are applied based on the wound type and healing phase-for example, using PDGF for inflammatory wounds and EGF for the proliferation phase. Additionally, combination therapies should be explored to assess synergistic effects by integrating growth factors with other treatments, such as stem cell therapy. Lastly, personalized medicine is recommended to tailor treatment plans to individual patient factors, including underlying conditions that may affect growth factor expression and response.

## FUTURE PERSPECTIVE

Growth factors in wound healing play a pivotal role in tissue repair, with key factors such as PDGF, TGF-β, VEGF, FGF, and EGF regulate cellular activities essential for wound closure, angiogenesis, and tissue regeneration [141]. A comprehensive understanding of the functions of these growth factors can facilitate the development of targeted therapies to enhance wound healing [142]. Current research highlights significant gaps in the study of growth factors in wound healing. One such gap involves the interactions among growth factors. While individual factors like VEGF and PDGF have been extensively studied, their interactions and synergistic effects and interactions remain underexplored [143]. A deeper understanding of these interactions could lead to more effective therapeutic strategies. Furthermore, signaling pathways activated by growth factors during the wound-healing process are not fully elucidated. Mapping these pathways is essential, particularly in chronic wounds, which often exhibit dysregulated healing responses. Chronic wounds pose unique challenges due to persistent inflammation and impaired healing [144]. Research is needed to determine how growth factors can be effectively utilized or modified to address these specific conditions. Gene therapy offers a promising approach by enabling the delivery of growth factor genes directly to wound sites. This could enhance local production of these proteins, potentially improving healing outcomes. Additionally, this approach allows for personalized treatment strategies tailored to individual patient needs. The development of bioengineered growth factors also holds significant potential. Creating more stable biological active versions of these factors can improve their efficacy in promoting wound healing [145]. Nanotechnology-based targeted delivery systems further enhance this approach, allowing controlled and sustained release of growth factors. Advanced clinical trials focusing on personalized medicine in wound care are urgently needed. These trials should evaluate the effectiveness of combining growth factors with other therapies, such as stem cell treatments or novel biomaterials. Several emerging technologies are also poised to transform wound healing. 3D Bioprinting technology can create complex tissue structures that incorporate growth factors and cells, potentially leading to more effective repair strategies. 3D bio-printed scaffolds designed to mimic the natural extracellular matrix provide a supportive environment conducive to healing. Similarly, cell therapies combined with growth factor treatments can significantly enhance tissue regeneration [146]. Stem cells, capable of secreting various growth factors, create a synergistic effect that promotes healing more effectively than either approach alone. The development of interactive dressings equipped with sensors for real-time monitoring of wound conditions represents a significant advancement [147]. These dressings can release growth factors or other therapeutic agents in response to specific stimuli, optimizing the healing process [148]. Such innovations, when combined with a deeper understanding of growth factor biology, hold immense promise for advancing wound care and addressing the challenge of chronic and complex wounds.

## Figures and Tables

**Fig. (1):**
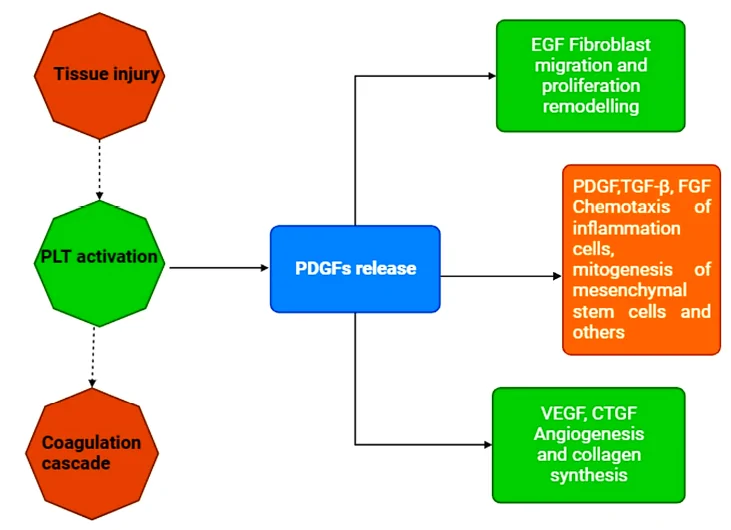
The role of platelet-derived growth factor (PDGF) in the process of repairing injuries. Platelet-derived growth factor (PDGF), epidermal growth factor (EGF), vascular endothelial growth factor (VEGF), connective tissue growth factor (CTGF), transforming growth factor-β (TGF-β), and fibroblast growth factor (FGF), Platelet Count (PLT) are among the terms used.

**Fig. (2):**
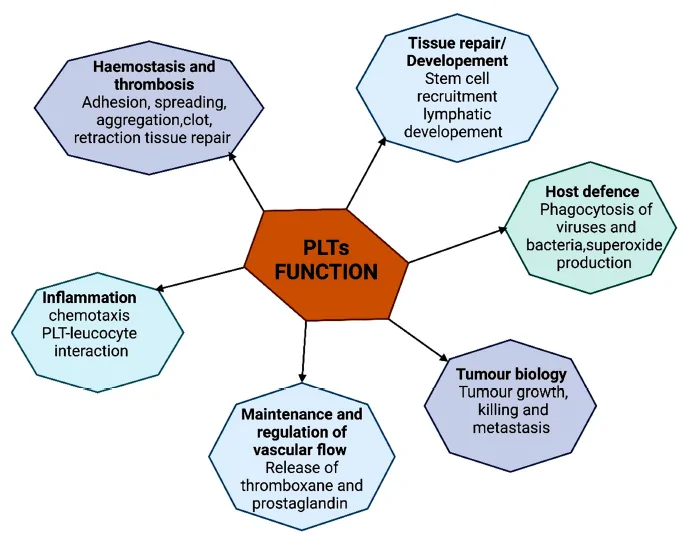
Platelets' functional responsibilities (PLT).

**Fig. (3):**
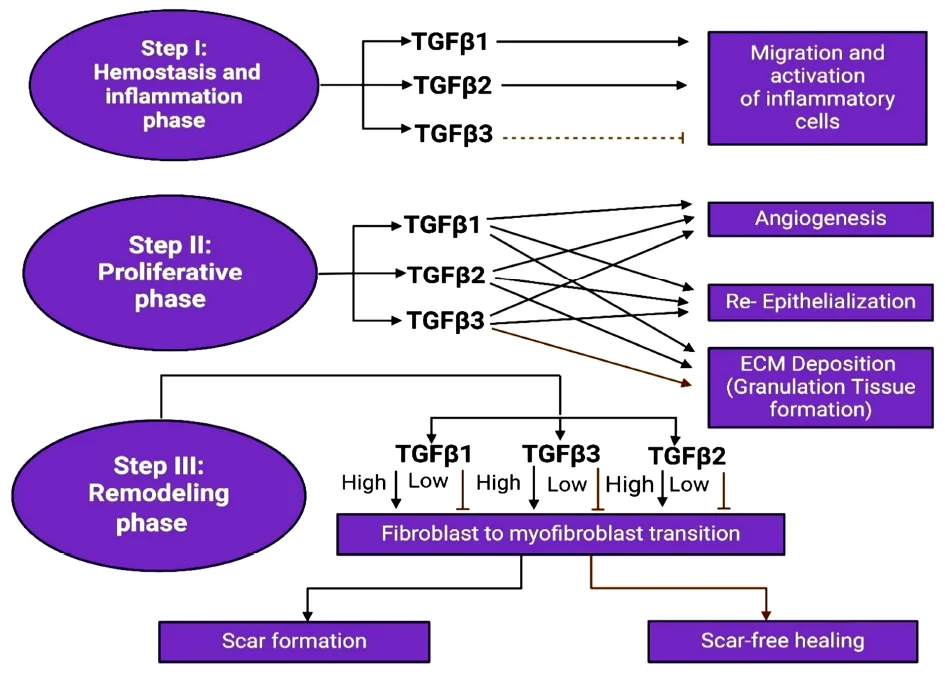
TGF isoforms are involved in the cutaneous wound-healing process.

**Fig. (4):**
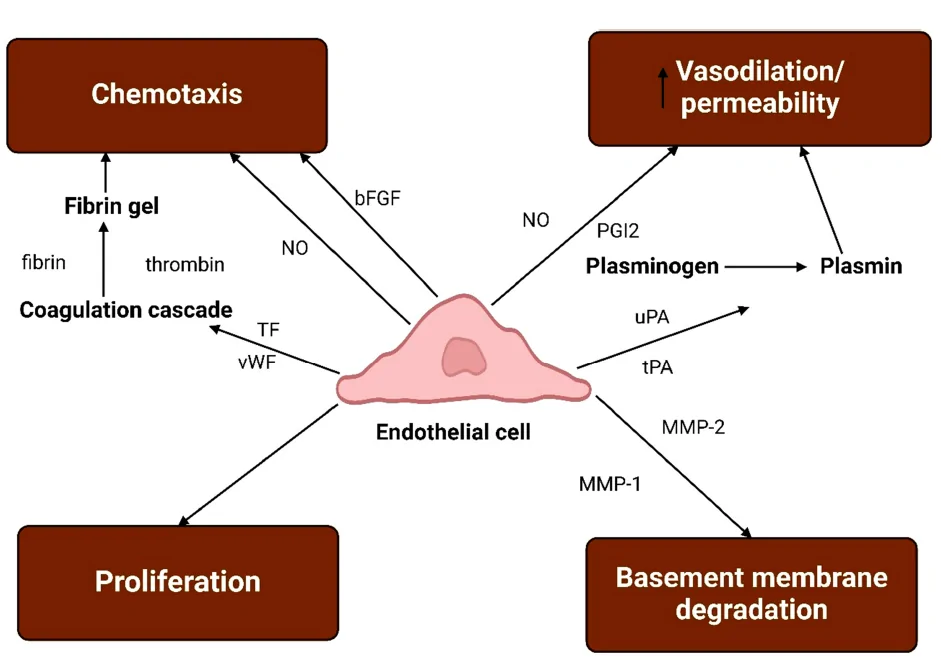
An illustration of the possible role of VEGF in wound healing. Several stages of the angiogenesis cascade escalate by activating the arterial cell. This illustration shows the synergistic angiogenesis-promoting factors that might arise from applying VEGF to a wound.

**Fig. (5):**
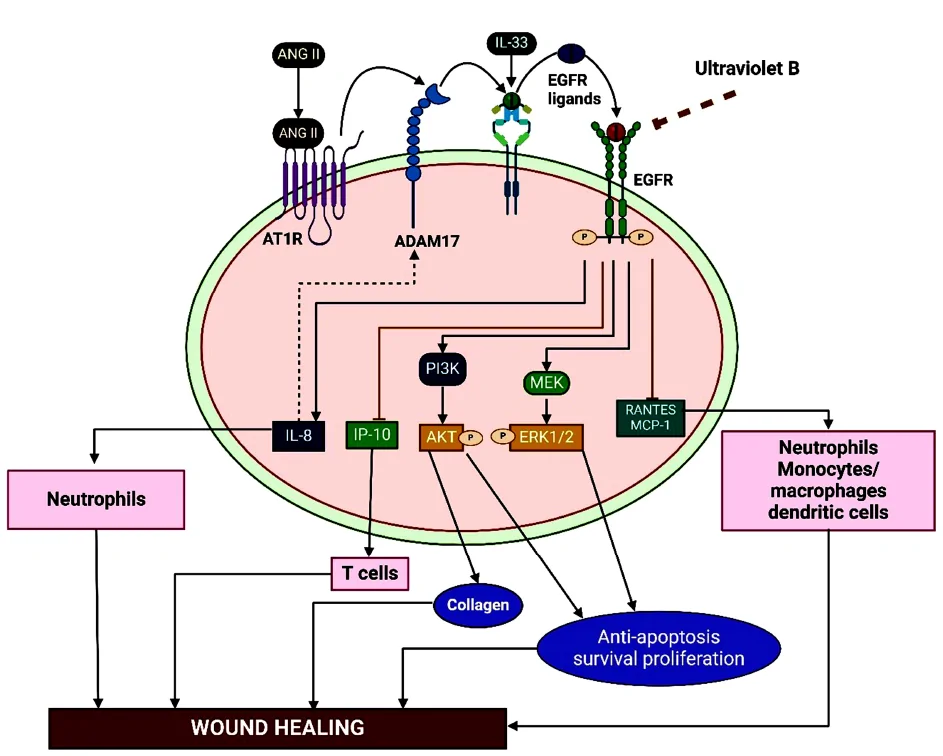
Skin wound healing and EGFR activation or transactivation.

**Fig. (6):**
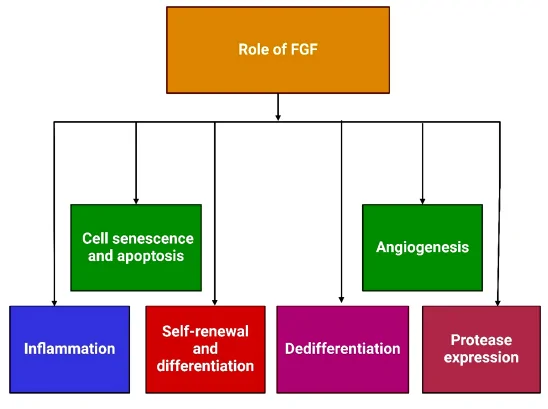
Function of FGF in processes of tissue healing.

**Fig. (7):**
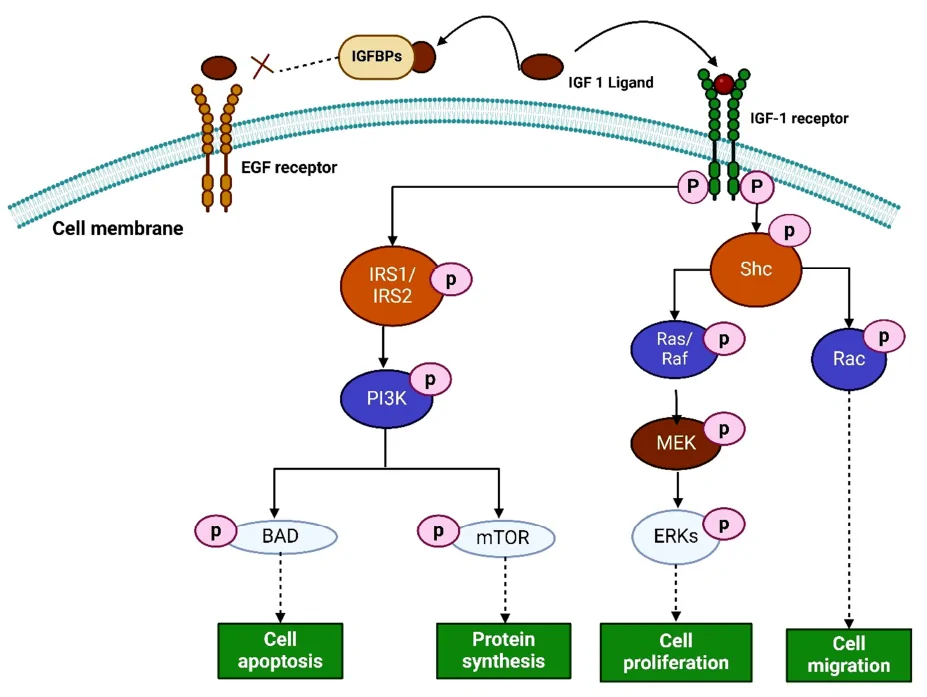
IGF-1, or insulin-like growth factor 1, activates the signal transduction cascade-connected to the insulin-like growth factor type 1 receptor (IGF1R). The IGF-1 receptor, also known as IGF1R, is activated, which initiates many signaling pathways.

**Fig. (8):**
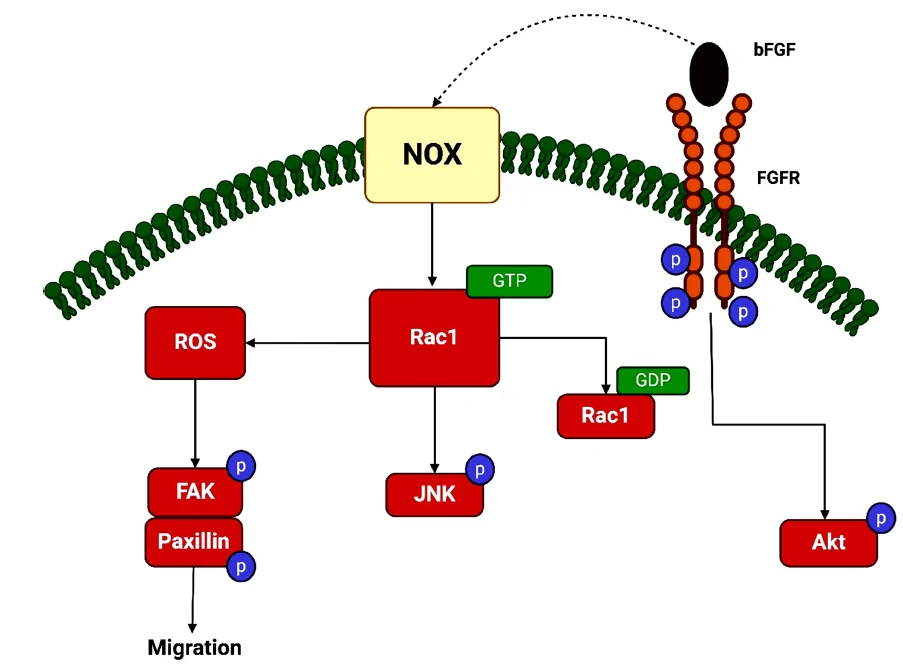
The role of bFGF signaling in stimulating migration in human fibroblasts to the skin by triggering PI3K/Akt-Rac1-FAK-JNK signaling, which results in the cytoskeletal remodeling necessary for fibroblast migration.

**Fig. (9):**
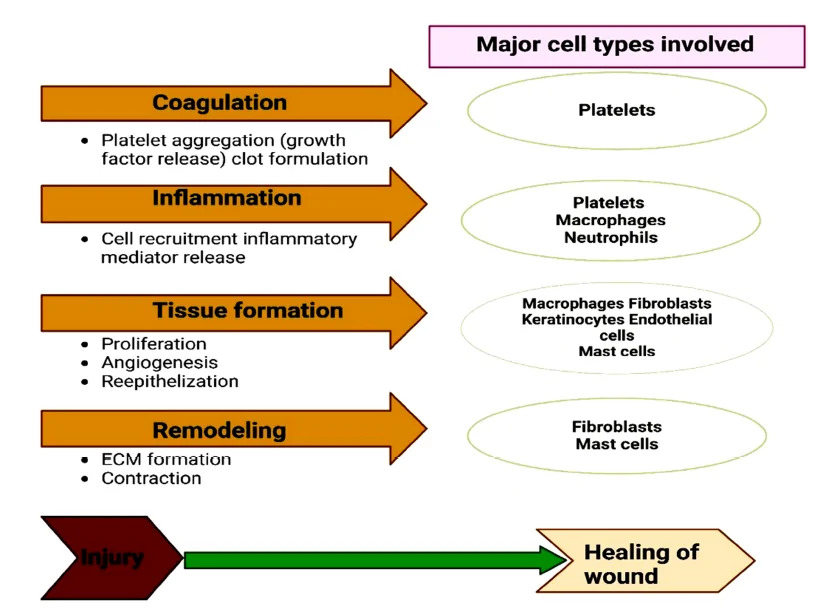
A wound healing processes.

**Fig. (10):**
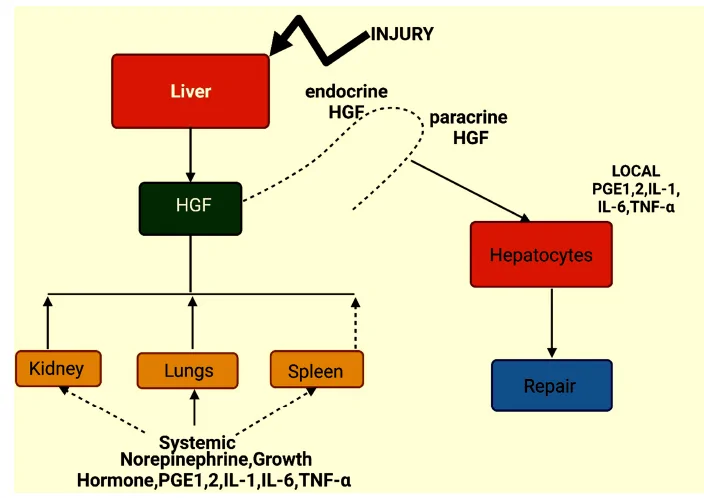
The HGF-c-Met pathway plays a major role in liver regeneration. During liver injury, additional organs, including the kidney, lung, and spleen, produce HGF in response to HGF-inducers such as norepinephrine, growth hormone, prostaglandins, and cytokines (*i.e.*, the endocrine system). Furthermore, HGF is synthesized by sinusoidal cells, or the paracrine system, including Kuppfer cells. Both processes are required for compensatory liver success and *in situ* repair.

**Fig. (11):**
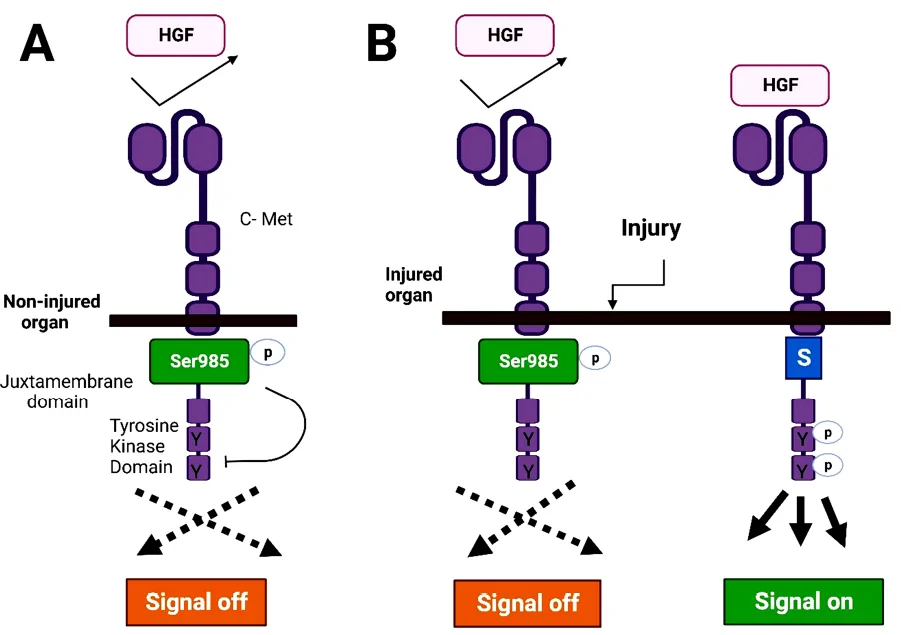
A theoretical representation of intracellular HGF/c-Met signaling in response to damage. (**A**) In non-damaging or normal organ tissues, c-Met's juxtamembrane has a serine residue site at position 985 (Ser-985) that is constitutively phosphorylated and acts as a negative regulator to block HGF-Met signaling, or turn it off. (**B**) Ser-985 site is “de-” phosphorylated after damage to organ tissues, most likely as a result of intracellular PP2A recruitment. Thus, phosphorylation of the c-Met tyrosine sites results in the delivery of pleiotropic actions.

## LIST OF ABBREVIATIONS

ADAM: A Disintegrin and Metalloprotease 12
ADP: Adenosine Diphosphate
AKT: Ak Strain Transforming (Protein Kinase B)
BFGF: Basic Fibroblast Growth Factor
cGMP: Guanosine 3',5'-cyclic monophosphate
CTGF: Connective Tissue Growth Factor
DNA: Deoxyribonucleic Acid
ECM: Extracellular Matrix
EGF: Epidermal Growth Factor
FGF: Fibroblast Growth Factor
GLUT: Glucose Transporters
HGF: Hepatocyte Growth Factor
IGF: Insulin-like Growth Factor
INSR: Insulin Receptor
KDR: Kinase Insert Domain Receptor
KGF: Keratinocyte Growth Factor
MAPK: Mitogen Activated Protein Kinase
MMPs: Matrix Metalloproteinases
NGF: Nerve Growth Factor
OPN: Osteopontin
PC: Platelet Concentrate
PDGF: Platelet-derived Growth Factor
PKC: Protein Kinase C
PLC: Pityriasis Lichenoides Chronica
PLT: Platelet Count
PRP: Platelet Rich Plasma
RAS: Renin Angiotensin System
RNA: Ribonucleic Acid
SDS: Sodium Dodecyl Sulfate
TGF-β: Transforming Growth Factor-beta
VEGF: Vascular Endothelial Growth Factor
VPF: Vascular Permeability Factor
VWF: Von Willebrand Factor
